# A Handheld Point-of-Care Genomic Diagnostic System

**DOI:** 10.1371/journal.pone.0070266

**Published:** 2013-08-01

**Authors:** Frank B. Myers, Richard H. Henrikson, Jennifer Bone, Luke P. Lee

**Affiliations:** 1 Department of Bioengineering, University of California, Berkeley, California, United States of America; 2 Berkeley Sensor and Actuator Center, University of California, Berkeley, California, United States of America; 3 Department of Physics, University of California, Berkeley, California, United States of America; McGill University Health Centre, McGill University, Canada

## Abstract

The rapid detection and identification of infectious disease pathogens is a critical need for healthcare in both developed and developing countries. As we gain more insight into the genomic basis of pathogen infectivity and drug resistance, point-of-care nucleic acid testing will likely become an important tool for global health. In this paper, we present an inexpensive, handheld, battery-powered instrument designed to enable pathogen genotyping in the developing world. Our Microfluidic Biomolecular Amplification Reader (µBAR) represents the convergence of molecular biology, microfluidics, optics, and electronics technology. The µBAR is capable of carrying out isothermal nucleic acid amplification assays with real-time fluorescence readout at a fraction of the cost of conventional benchtop thermocyclers. Additionally, the µBAR features cell phone data connectivity and GPS sample geotagging which can enable epidemiological surveying and remote healthcare delivery. The µBAR controls assay temperature through an integrated resistive heater and monitors real-time fluorescence signals from 60 individual reaction chambers using LEDs and phototransistors. Assays are carried out on PDMS disposable microfluidic cartridges which require no external power for sample loading. We characterize the fluorescence detection limits, heater uniformity, and battery life of the instrument. As a proof-of-principle, we demonstrate the detection of the HIV-1 *integrase* gene with the µBAR using the Loop-Mediated Isothermal Amplification (LAMP) assay. Although we focus on the detection of purified DNA here, LAMP has previously been demonstrated with a range of clinical samples, and our eventual goal is to develop a microfluidic device which includes on-chip sample preparation from raw samples. The µBAR is based entirely around open source hardware and software, and in the accompanying online supplement we present a full set of schematics, bill of materials, PCB layouts, CAD drawings, and source code for the µBAR instrument with the goal of spurring further innovation toward low-cost genetic diagnostics.

## Introduction

Integrated microfluidic diagnostic systems present an unprecedented opportunity to facilitate high-quality, low-cost healthcare in remote and resource-limited settings [1]. These systems show promise for both acute infection discovery and chronic disease monitoring and management [2]. In particular, a wide array of nucleic acid tests (NATs) are emerging to identify pathogen species as well as specific clinically-relevant characteristics such as pathogenicity, origin, and drug susceptibility [3], [4]. The importance of rapid diagnostics for infectious diseases is highlighted by the threat of increasing drug resistance due to indiscriminate treatment where a rapid test could improve patient outcome and reduce the emergence of more deadly strains [5], [6].

Although developed economies have access to more accurate molecular diagnostics, including polymerase chain reaction (PCR) tests, these tests have yet to be translated into diagnostics that meet the specific needs and requirements of point-of-care testing. Lateral flow immunoassays have made a significant impact in the diagnosis of diseases like HIV, but it is difficult to adapt this assay format to situations where a quantitative readout or molecular amplification is required. Recent efforts have focused on automating standard sample preparation and PCR techniques; however the significant assay cost and relatively low throughput remain prohibitive barriers for wide-scale adoption [7].

Recently, several methods have been developed to enable isothermal amplification, facilitating reduced power consumption and expanded options for material and chemical selection [8]. Some leading isothermal nucleic acid amplification techniques include Loop-Mediated Isothermal Amplification (LAMP) [9], Recombinase Polymerase Amplification (RPA) [10], and Rolling Circle Amplification (RCA) [11], among others. Furthermore, these methods frequently offer improved performance over standard PCR, achieving highly sensitive and specific results in as little as 15 minutes. LAMP may be particularly well-suited for blood-based detection because Bst polymerase is not inhibited by hemoglobin, whereas Taq polymerase used in PCR is [12]. This may allow LAMP to work with much “dirtier” samples. Furthermore, the amount of DNA produced by LAMP (and hence the fluorescence signal) is considerably higher than that produced by PCR, allowing it to be used with inexpensive detection hardware.

A number of recent efforts have aimed to miniaturize isothermal amplification diagnostic assays for use in resource-poor settings [8], [13]. However, no isothermal amplification system yet provides sample-in, answer-out capability. All of these systems presented to date work with conventional PCR tubes which limits the degree to which they can be expanded to include multi-step assays including sample prep and multiplexed detection. Microfluidic assay cartridges offer many compelling advantages over conventional tubes, most notably the degree to which multiple assay steps can be incorporated on one integrated device. Our goal in the present work is to introduce a platform for isothermal amplification and real-time detection on a multiplexed microfluidic cartridge. Such a cartridge could be coupled with some of the microfluidic sample preparation techniques already presented [14], [15], yielding sample-in, answer-out diagnostic at a significantly reduced cost as compared with PCR.

Loop-mediated isothermal amplification (LAMP) has been adapted for a range of assays, including infectious pathogen detection and genotyping of single nucleotide polymorphisms (SNPs) [16]. There has recently been interest in leveraging this assay in a miniaturized format, however a complete integrated system for multiplexed sample analysis has yet to be established. Fluorescent analysis offers a number of advantages over competing optical or electrochemical detection methods, however implementing sensitive fluorescence detection inexpensively remains a challenge [17]. We have developed a microfluidic biomolecular amplification reader (µBAR), coupled with a disposable assay cartridge, for efficient and inexpensive disease analysis compatible with isothermal amplification techniques [18]. We have characterized the performance of the µBAR system and conducted initial tests for LAMP-based detection of HIV (via the *integrase* gene).

## Materials and Methods

### A. Microfluidic Cartridge

**Figure 1:**
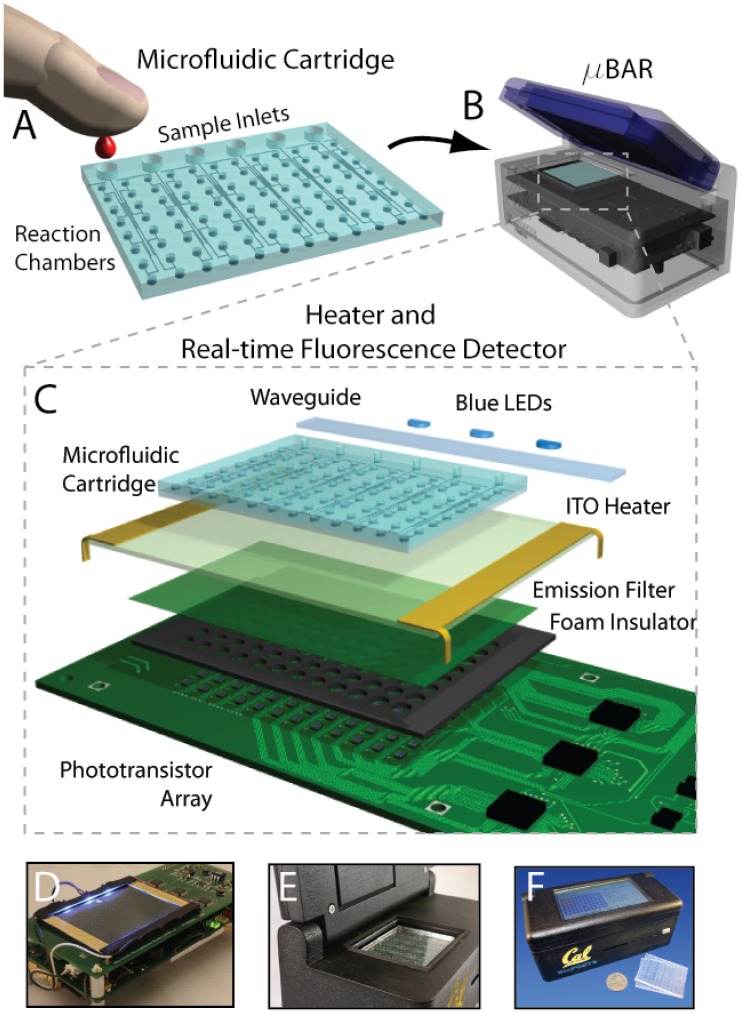
The design of the µBAR system and operation. (A) A disposable microfluidic cartridge is loaded with sample fluid (e.g. blood) via SIMBAS degas-driven flow. This technique proceeds automatically without any pumping or external power. LAMP reaction mix (primers, enzymes, dNTPs, etc.) may be lyophilized on-chip or mixed with the sample before loading. After 30 min, the chip is fully loaded and is placed in the µBAR instrument. (B–C) The instrument features blue excitation LEDs to the side of the chip. Waveguides help to ensure the light is efficiently coupled to the PDMS chip without stray scattering. An ITO substrate provides uniform heating across the reaction chambers of the chip. Below this substrate is a green plastic emission filter following by a neoprene foam barrier with holes which optically isolate each phototransistor on the PCB underneath. The neoprene provides both thermal and optical insulation to the system. The microfluidic chip sticks to the surface of the ITO via van der Waals forces, maximizing thermal transfer and ensuring that the chip does not move during heating. (D) µBAR with case removed, showing side LED illumination through waveguides. (E) µBAR with chip inserted. (D) Assembled handheld µBAR instrument and microfluidic cartridges with a US quarter for size comparison.

We have developed a disposable microfluidic device which passive degas-driven fluid actuation (Figure 1A) [14]. The chip measures 55×42×5 mm and consists of 96 parallel reaction chambers at 4.5 mm pitch supplied by 6 separate sample inlets for multiplexed analysis. Each circular reaction chamber is 2 mm in diameter and 200 µm in height, for a total volume of 628 nL.

The chambers are cast in polydimethylsiloxane (PDMS) using standard soft lithography techniques [19] and bonded to a thin transparent elastomer base using oxygen plasma [20]. The chip is UV sterilized for 24 hours after bonding. After bonding, the device is exposed to a vacuum of ∼300 mTorr for >1 hour, and then vacuum sealed using a commercial food sealer. Due to the elastomeric pore structure of the PDMS, the chip will begin drawing in sample introduced into the inlets immediately upon exposure to atmospheric pressure. Samples can be completely loaded in less than 1 hour (see Movie S1). This time can be significantly reduced with smaller reaction chambers. Chips can be stored vacuum sealed for at least one month (likely much longer) without loss of vacuum. This degas-driven loading method enables us to use dead-end reaction chambers which help ensure that amplicons remain on chip and reduce the risk of contamination between amplification runs. Although we have used the LAMP method in the design of these primer sets, this chip is compatible with other nucleic acid amplification techniques.

### B. LAMP Assay

We have adapted the calcein metal indicator fluorophore as a tool for both real-time fluorescence measurement and naked-eye readout of signal based on initial target presence. Briefly, this method involves the use of a calcein dye that is initially quenched by manganese ions. As a byproduct of nucleic acid amplification, pyrophosphate groups are produced in abundance and readily precipitate out of solution as manganese pyrophosphate, removing the quencher from calcein and yielding a bright fluorescent signal [21].

For our proof-of-principle amplification assay, all reagents were mixed under sterile conditions immediately prior to loading them on the chip. A total reaction volume of 20 µL was loaded into each inlet on the chip (this gets distributed among 16 reaction wells). The same sample was simultaneously used with the PCR thermocycler (20 µL reaction volume). The reaction mix consisted of: 1.6 µM each of FIP/BIP primers, 0.8 µM each of Loop-F/Loop-B primers, 0.2 µM each of F3/B3 primers, 27.3 µM Calcein, 0.8 µM Bst DNA Polymerase, 1.4 mM dNTPs, 20 mM Tris Buffer, 10 mM KCl, 8 mM MgSO_4_, 1 mM (NH_4_)_2_SO_4_, 1 mM Tween-20, 0.8 M Betaine, and 1.49 mM MnCl_2_. The sample template (purified DNA) was added last.

### C. Instrument

**Figure 2:**
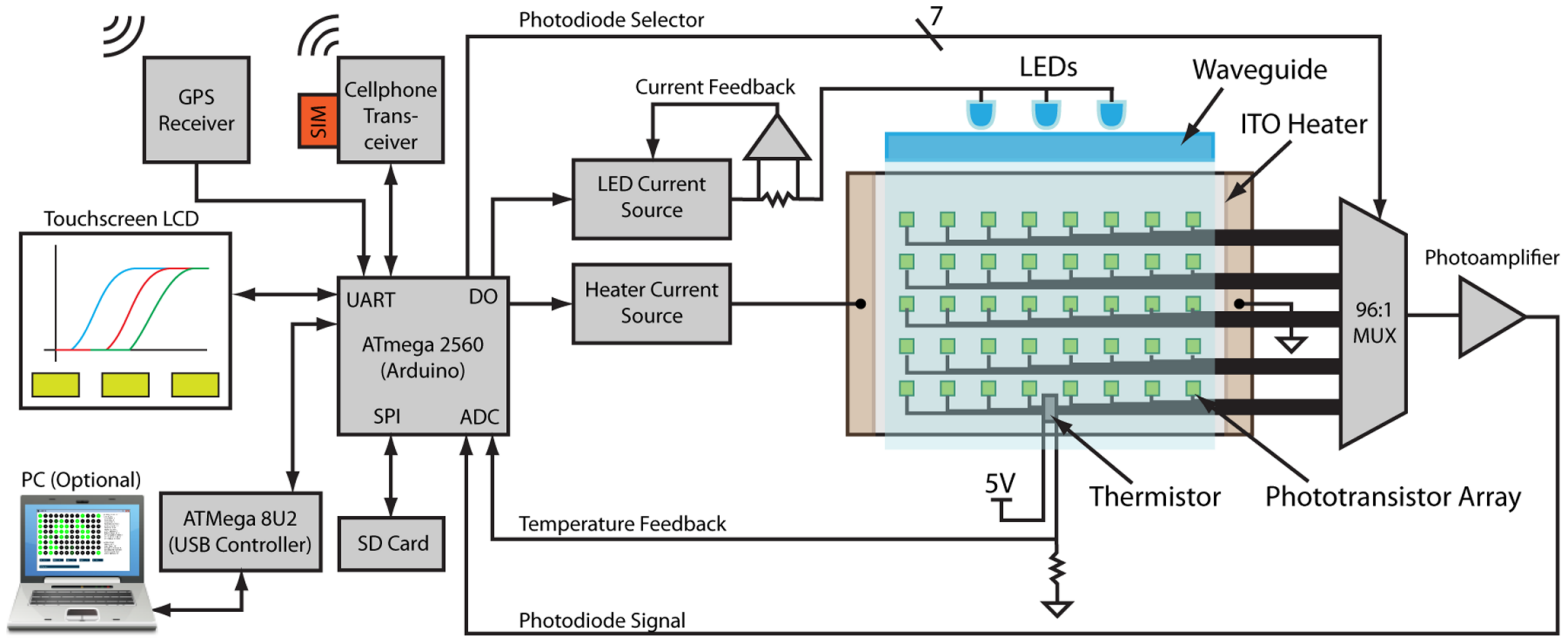
Block diagram of the µBAR system. The system is controlled by an ATmega 2560 microcontroller which has been loaded with the Arduino bootloader firmware. A GPS receiver and cell phone transceiver facilitate remote field diagnostics and epidemiological studies. The system can receive user input via either an LCD touchscreen or PC via USB. Data is stored to an SD card and also broadcast to the PC, if connected. A monolithic LED driver supplies a constant current to 3 blue InGaAs LEDs (even as battery voltage decreases), and a booster circuit delivers a constant current to the ITO heater. The microcontroller receives temperature feedback from a thermistor and turns the heater on and off accordingly. An array of 96 phototransistors are positioned underneath the heater and an analog multiplexer is used to raster across the array and read photocurrents from each location using a single photoamplifier.

For quantitative readout of the assay, the chip is inserted into a battery-powered instrument which maintains assay temperature, illuminates the chip, and detects fluorescence emission from the reaction chambers using an array of phototransistors (Figure 1C–F). It does this without the use of costly optical components, and without the need for alignment or focusing. The instrument is automated with an Atmel Atmega2560 microcontroller implementing the Arduino bootloader and USB interface. The instrument features a Secure Digital (SD) Flash memory card reader for storing assay parameters and results, and a 4.3 inch color touchscreen user interface (Amulet Technologies). Additionally, the instrument includes a GSM cell phone module and a GPS module, thus enabling epidemiological surveillance and medical coordination in remote locations. A block diagram is provided in Figure 2.

To run an assay, the microfluidic chip is inserted directly on top of a 47×67 mm indium tin oxide (ITO) coated glass slide which heats the chip to 60°C. The ITO is connected to a TPS61085 boost converter which delivers 1 A through the slide. The chip reaches its set point temperature in less than 20 minutes. The temperature is maintained by toggling the boost circuit with a solid state relay. A thermistor in a half-bridge circuit configuration provides temperature feedback. This thermistor is mounted directly underneath the ITO slide. For LAMP and other isothermal reactions, the temperature is held constant, whereas for PCR it can be programmed to cycle.

Three blue InGaN LEDs (peak = 472 nm) illuminate the chip from the top side through rectangular glass waveguides cladded with black paint to minimize stray light. The waveguides promote total internal reflection (TIR) of excitation light within the chip. The refractive index of glass, PDMS, and water are similar enough that reflections of excitation light off of internal surfaces of the chip do not significantly contribute to background signal on the phototransistors. The LEDs are driven by a second boost circuit based on the MIC3289 which delivers a controlled current to the LEDs which does not vary with battery charge state. This chip provides 16 logarithmically-spaced intensity levels which are digitally selected.

**Figure 3:**
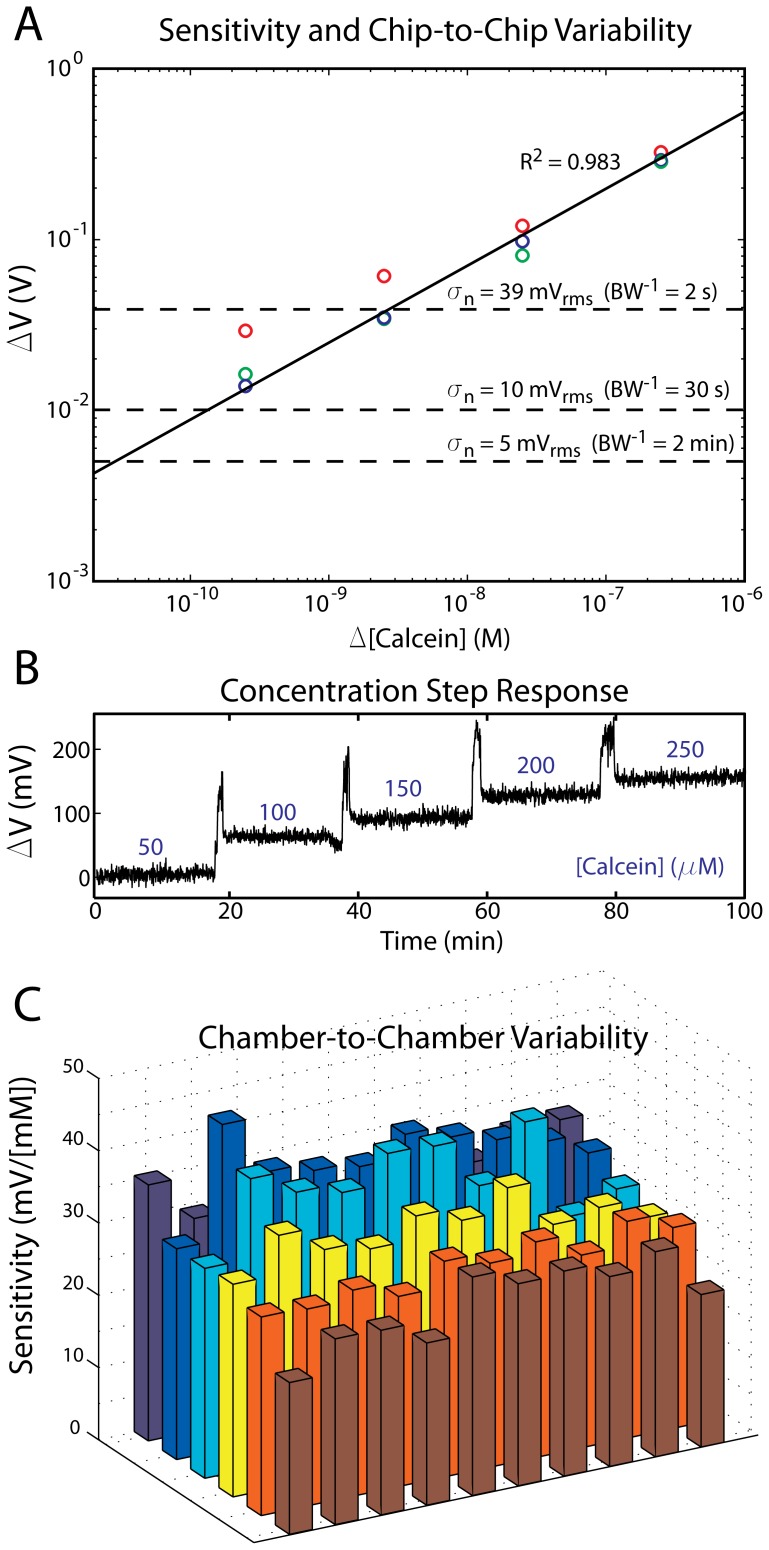
Fluorescence sensitivity and linearity. (A) Different concentrations of calcein were introduced into a microfluidic cartridge and the corresponding change in photoamplifier voltage was recorded. The circles indicate the mean signal amplitude across all reaction wells for three different chips (red, green, and blue). As expected, the relationship between calcein concentration and photoamplifier output is linear (R^2^ = 0.983). Limit of detection can be evaluated by determining when this trend line crosses the noise floor of the instrument at a given bandwidth. At a time resolution (BW^−1^) of 2 min, the µBAR can distinguish changes in calcein concentration of 30 pM, which is orders of magnitude below the fluorophore concentrations typically used in nucleic acid amplification reactions. (B) 50 µM steps of calcein are clearly visible in the photoamplifier output and this output remains stable for a constant concentration. (C) Due to the nature of the illumination scheme and the lack of optics, there is some nonuniformity of sensitivity across the 6×10 reaction chamber array. However, we take this into account and normalize each well by its mean sensitivity, shown here. In this graphic, the illumination LEDs and sample inlets are located above the topmost row.

The calcein used in the LAMP reaction emits green fluorescence (peak excitation wavelength = 480 nm, peak emission wavelength = 515 nm). This fluorescence is detected with a phototransistor located directly underneath each chamber. Importantly, there is a small air gap between the phototransistor housings and the ITO heater, which ensures TIR and reduces feedthrough of the excitation light into the phototransistors. Each one of the 96 phototransistor is wired to one of three 32∶1 analog multiplexers (AD732). The microcontroller uses these multiplexers to raster through the phototransistor array, selecting one at a time for interrogation. The entire array is sampled at a specified interval (2 seconds, typically). The outputs of the multiplexers are connected to a transimpedance amplifier with an input biased to 2.5V. When a phototransistor is selected, this bias voltage allows collector current to flow in proportion to the illuminance on the phototransistor’s surface. Phototransistors, rather than photodiodes, were chosen because the leakage current of CMOS analog multiplexers is significant compared to photodiode currents leading to crosstalk problems. Phototransistors provide significantly more current for the same illuminance and in this configuration they are be biased through the multiplexer which eliminates the possibility of crosstalk. The photocurrent from each phototransistor is sampled over time and recorded to the SD card or to an attached PC via USB. To render amplification plots, the raw data is first baseline subtracted. The baseline is estimated based on a linear regression of the raw data from the first 10–20 minutes of the assay run (it is assumed that no amplification occurs before 20 minutes). The signal from each well is then normalized by that well’s predetermined sensitivity coefficient (Figure 3C). Finally, the signal is smoothed with a moving average span of 100 samples to improve SNR.

The instrument is powered by a 3.7 V, 2000 mAh lithium polymer battery. A third boost converter (LTC3525) delivers a 5V supply to the digital electronics, LCD display, and transimpedance amplifier. A typical assay run lasts approximately 2 hours and consumes 500 mAh. The heater dominates power consumption. Our ultimate goal is to create a fully-integrated, portable instrument which addresses the needs of remote/resource poor settings. The specific components included can be tailored for the situation. At its simplest, this instrument would be a handheld heater and LED illuminator which would allow qualitative naked eye readout.

## Results

### A. Fluorescence Sensitivity

We first characterized the fluorescence sensitivity of the µBAR instrument by introducing different known concentrations of calcein (the fluorophore used in our LAMP assays) into the microfluidic device and observing the relative changes in photoamplifier output across all reaction chambers (Figure 3A). For this experiment, we developed a slightly modified chip which included a serpentine channel connecting all of the reaction wells in series. This allowed us to sequentially introduce higher calcein concentrations (Figure 3B) while ensuring that the chip placement/geometry was fixed. We repeated this experiment with 3 independent chips (marked with red, blue, and green circles) and found that detector output as a function of concentration followed a linear trend over four orders of magnitude. To determine the sensitivity of the instrument to fluorescence changes, we calculated the Instrument Detection Limit (IDL) as the concentration that produces a signal three times greater than the noise standard deviation of the instrument for a given time resolution. In the case of realtime nucleic acid amplification signals, a time resolution (BW^−1^) of 2 min is sufficient. In that case, the noise standard deviation of the µBAR is 5 mV_rms_, leading to an IDL of approximately 600 pM with the µBAR. As most nucleic acid amplification reactions involve fluorophore concentrations that are orders of magnitude higher than this (typically 1–10 µM), this is more than sufficient to carry out these kinds of assays. We further examined the nonuniformity of photoamplifier sensitivity across the detection array. Because the LED lighting does not lead to uniform illumination, and due to some internal scattering within the chips, the amount of light reaching each reaction chamber is slightly different. By calculating the linear slope of amplitude change as a function of concentration change (i.e. sensitivity) at each well (Figure 3C), we can normalize subsequent assay runs so that intensities can be directly compared across the chip. Although the chip was designed with 96 reaction chambers in a 12×8 configuration, we determined that it was not possible to use assay results from the outermost chambers due to excitation light scattering at the edges of the device. This limitation could easily be overcome in subsequent designs (see discussion). We therefore restrict our analysis to the 60 innermost reaction chambers (6×10 array).

### B. Heater Performance

**Figure 4:**
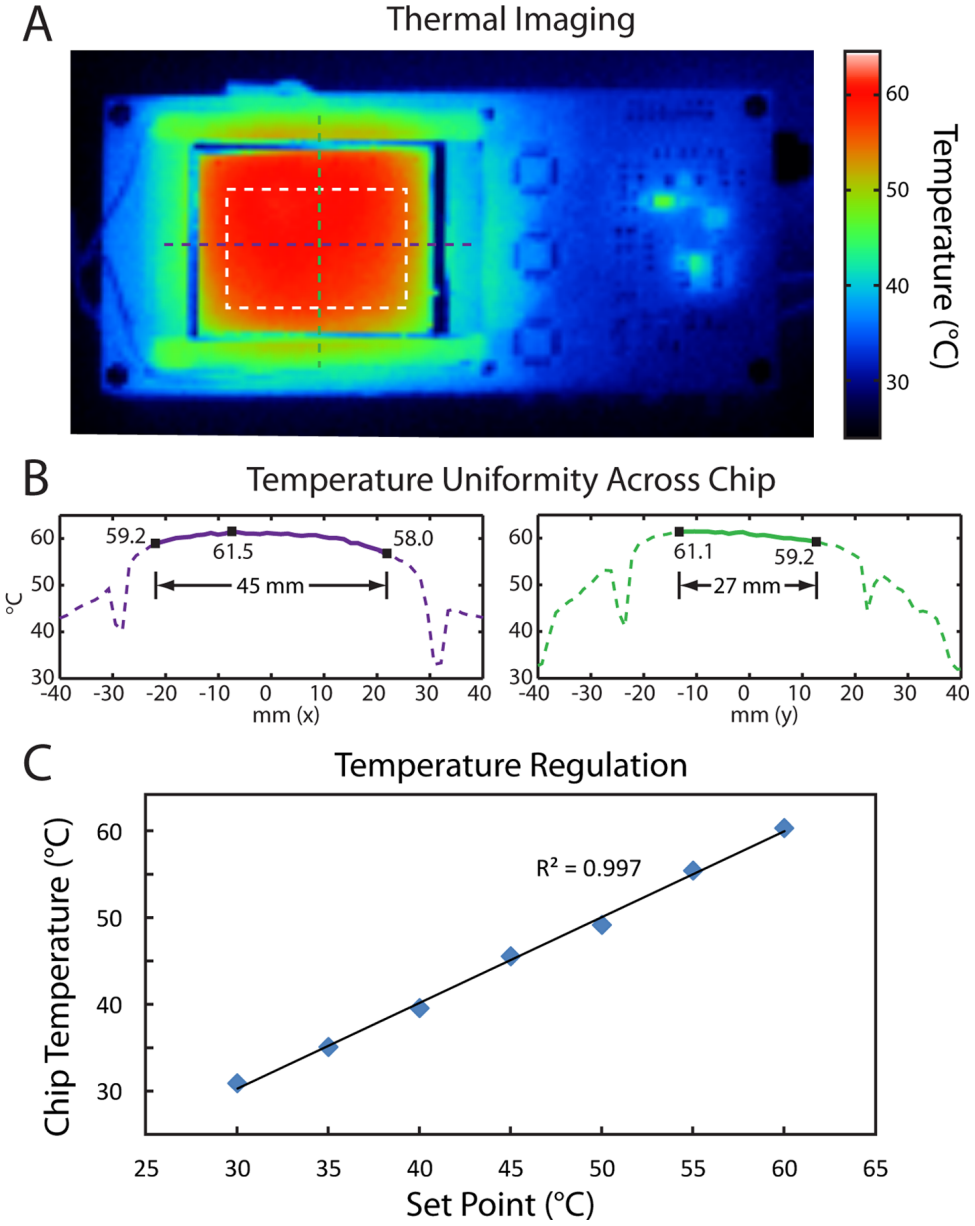
Heater uniformity and stability. (A) Infrared thermal image of the µBAR (case removed) at 60°C. The white box indicates the extent of the 6×10 reaction chamber array. (B) Linescans through this region indicate a temperature uniformity of ±1.25°C. (C) Chip temperature, as measured with a thermocouple directly embedded in the PDMS, versus µBAR temperature set point.

We next examined the spatial uniformity and temperature regulation performance of the µBAR’s internal ITO substrate heater. We used an infrared thermal camera to examine the surface of a PDMS device on top of the ITO heater with the case removed (Figure 4A). For the 6×10 array of reaction chambers in the center of the chip, we found a temperature distribution of 60±1.25°C (see linescans, Figure 4B). By comparison, modern mid-range thermocyclers have a well-to-well temperature nonuniformity of 1–2°C during a plateau phase [22]. We then embedded a commercial thermocouple temperature probe (Fluke 87V) within the PDMS microfluidic device and calibrated the on-board temperature feedback (from a thermistor positioned on the underside of the ITO heater) with the actual chip temperature. Following calibration, we found excellent agreement between the µBAR’s temperature setpoint and the independently measured chip temperature (Figure 4C).

### C. Battery Life

**Figure 5:**
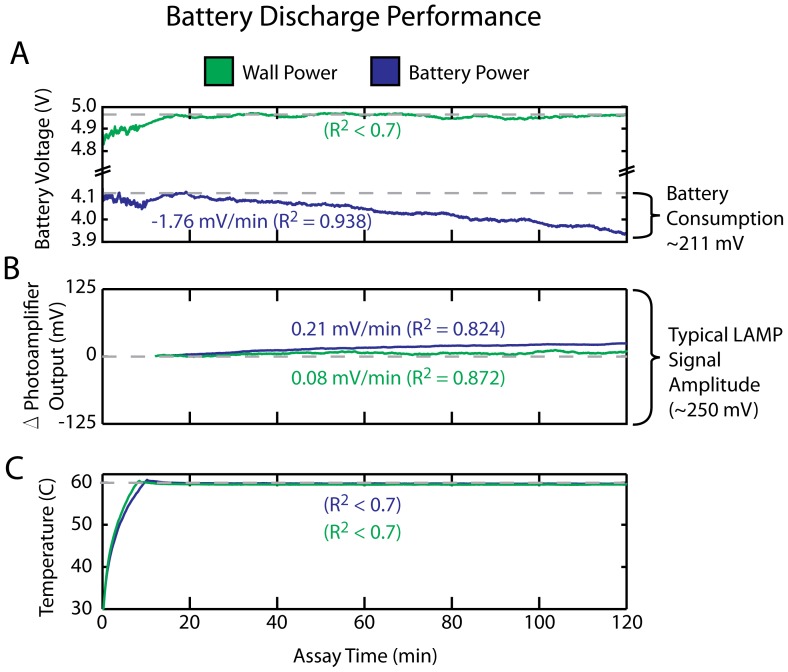
Battery discharge performance of the µBAR. The µBAR maintains constant LED intensity and chip temperature over a 2 hr period when operated from a battery. Since a battery’s voltage decreases as it is discharged, we wanted to ensure that this would not obscure results of the assay by either causing drift in the photoamplifier output or chip temperature. In this experiment, the battery voltage decreases linearly by 211 mV over the course of the assay run (A). This, however, does not significantly impact the intensity of the LEDs/photoamplifier sensitivity (B), or temperature stability (C), as compared with a constant (wall) power source. Dashed lines show the values of battery voltage, photoamplifier output, and temperature at t = 20 min, linear regressions are calculated and slopes are reported for R^2^ values >0.7. There is no significant change in temperature for either wall or battery power. Photoamplifier output shows a slight time dependant drift in both cases (7.5 mV/min for wall power and 2.5 mV/min for battery power), but this drift is significantly lower than the signal amplitudes which we see in a typical LAMP assay (∼250 mV, see fig. 6), so we consider it acceptable.

**Figure 6:**
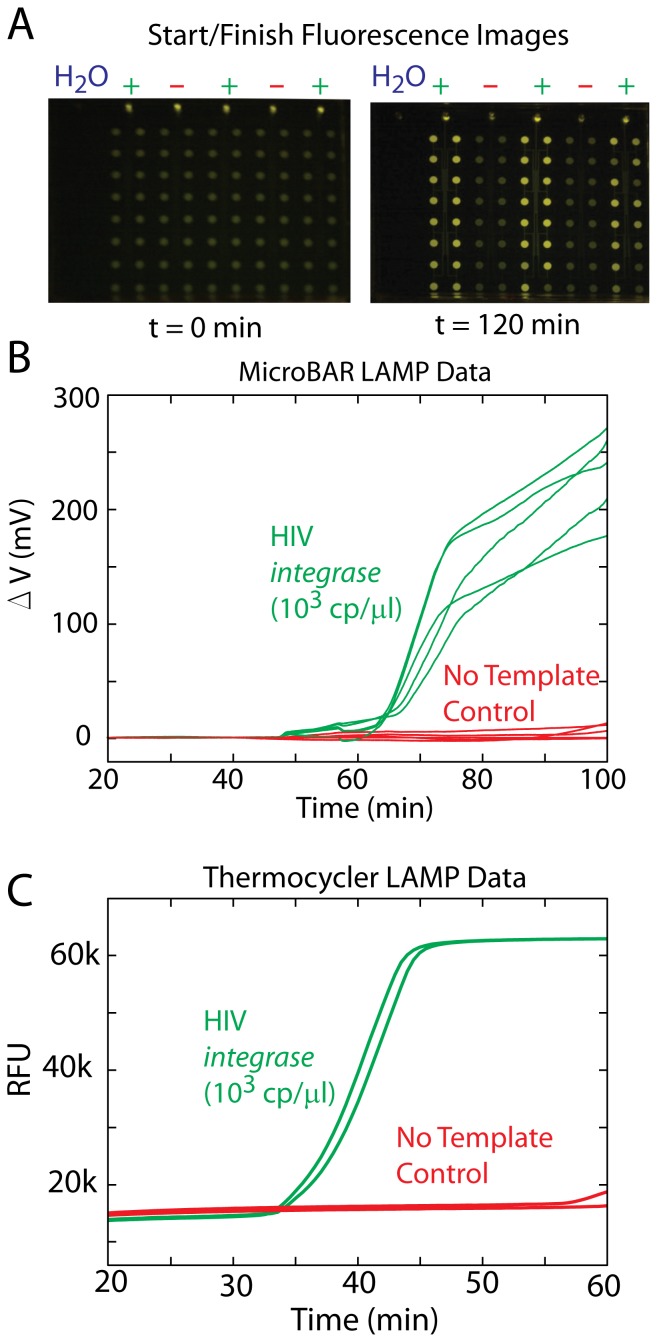
µBAR LAMP vs. **Thermocycler LAMP.** Results of the LAMP reaction for detection of the HIV *integrase* gene using both the µBAR and a conventional thermocycler. (A) Presence of the HIV *integrase* gene target results in an increase in fluorescence from the reaction wells, as seen in these before and after chip photos. (B) Photoamplifier voltages from 5 independent reaction wells are displayed here, all showing a positive result after about 70 minutes. (C) The same assay run on a conventional thermocycler shows positive results slightly faster (40 minutes), possibly due to the differences in assay volumes and thermal transfer characteristics.

The µBAR can either be operated from a 5V DC wall power source or from a lithium polymer battery (3.7V nominal voltage). During battery operation, the battery discharge leads to a decreasing battery voltage, and it’s important that this not significantly affect LED intensity, photoamplifier sensitivity, or temperature over the course of a typical 2 hour assay run, because any time-varying signal might obscure the assay results. Using a chip with a constant dye concentration, we directly compared photoamplifier output and temperature for wall power and battery power (Figure 5). We found that both wall power and battery power lead to a small linear drift in photoamplifier output (0.21 and 0.08 mV/min, respectively) which was well below the amplitude of a typical LAMP assay (>10 mV/min, see Figure 6). Both wall power and battery power resulted in a steady temperature of 60C ±0.1 (standard deviation).

### D. LAMP Genetic Amplification Assay Performance

Figure 6 shows the results of a loop-mediated isothermal amplification (LAMP) assay for the HIV *integrase* gene using our microfluidic chip and electronic reader. Two independent samples are shown, one containing a plasmid with the target gene at a concentration of 10^3^ copies/µL, and the other containing no DNA (no template control, NTC). The chips were vacuum treated at 300 mTorr, samples were dropped onto the chip (20 µL per inlet), and the chip was placed into the µBAR instrument upon completion of sample loading. Figure 6A shows before and after pictures of the microfluidic device under fluorescent illumination (490 nm excitation with a long-pass filter). Amplification is clearly visible in the chambers with the target HIV *integrase* plasmid as compared with the NTC chambers. Figure 6B shows that within 70 minutes, the positive samples exhibit readily detectable signals above the NTC background. When run on a conventional quantitative PCR thermocycler (Bio-Rad CFX96), the amplification occurs somewhat earlier (around 40 minutes). This discrepancy in time may be due to the differences in reaction volumes and surface-to-volume ratios of the two assays (each on-chip chamber contains about 1.2 µL sample volume, whereas the full 20 µL is in one reaction tube on the thermocycler). LAMP reagents may be adsorbing to the PDMS channels as they enter the chip, leading to lower concentrations overall within the wells and a heterogeneity from well-to-well (note that the final intensity varies somewhat from well to well). More work needs to be done to find an effective surface treatment method which enables vacuum-driven loading but prevents adsorption. As with qPCR (which can also be quite heterogeneous from well to well), we recommend running assays in duplicate or triplicate with the µBAR to mitigate this heterogeneity. Despite these shortcomings, our results indicate that the µBAR is able to detect amplification.

### E. Cost Breakdown

The µBAR enables point-of-care genetic amplification assays at a fraction of the cost, weight, and size of a traditional thermocycler instrument. The µBAR’s portability, combined with its integrated GPS and cell phone connectivity, enable rapid field deployment and epidemiological surveying within a geographic region. A full cost breakdown is provided in the bill of materials accompanying this paper (Table S1 and Table S2 in File S1). Prototype instruments can be built for $919 in single unit quantities. At production quantities (>5000 units), the µBAR’s total materials and manufacturing costs are estimated to be $382, and this drops to $223 if the LCD, GPS, and cell phone module are excluded. By comparison, a typical quantitative thermocycler instrument is priced at $30,000 and is poorly suited for developing world applications. The disposable microfluidic device requires approximately $0.90 in materials cost (PDMS and vacuum seal bag), leading to a final estimated manufacturing cost of $1.44 per chip (including molding, bonding, and vacuum packaging). The cost of the LAMP reaction mix (including primers, polymerase, dNTPs, etc.) is approximately $0.02/µL, leading to a total reagent cost of $1.24 per chip (assuming all 96 reaction chambers are used). All 96 reactions could be devoted to one patient sample or could be divided up among several patients to reduce cost. It’s important to note that this figure does not include sample preparation costs.

## Discussion

While traditional laboratory instruments for genetic and protein diagnostics are relatively mature and reliable, these instruments are impractical in the developing world, where healthcare must often be delivered in remote settings with limited infrastructure and by people with limited training. The µBAR platform represents a step toward enabling point-of-care genotyping in the developing world. It improves on the state-of-the-art in genetic amplification instrumentation in its cost, operational simplicity, and portability. Our instrument could be conceivably priced ∼50× cheaper than conventional real-time thermocyclers. It does not require external power and at least 3 assays can be run before the internal battery requires recharging. It provides the capability to geotag genetic assay results and transmit these results over cell phone networks, which are ubiquitous even in the developing world [23]. As a more complete picture of the genetic basis for disease emerges, tools like the µBAR will be invaluable in delivering effective treatment, combating drug resistance, and identifying emerging threats and bioterrorism agents. One of the major difficulties in resource limited settings is in scheduling follow-up visits with patients who may not have reliable access to communication. Any point-of-care diagnostic instrument should deliver sample-to-answer turnaround in a matter of hours so that a diagnosis and treatment can be rendered within the same visit. A complete diagnostic procedure (including sample collection and preparation) could conceivably be performed using the µBAR in under three hours. Furthermore, several patients’ samples can be run in parallel (we have included 6 inlets on the current chip) if higher throughput is necessary.

We have chosen the LAMP assay because it has many advantages in developing world applications. In comparison to PCR, LAMP does not require as thorough a DNA purification step, and has even been shown to work with “raw” samples such as blood, urine, and saliva [24]. Because our microfluidic cartridge is completely sealed during manufacture and because samples are loaded into dead-end channels via degas driven flow, there is minimal risk that contaminating DNA could get in the chip before the sample is loaded, and there is also minimal risk of amplicons escaping the chip and contaminating the work environment after the assay has run. We have demonstrated excellent fluorescence sensitivity and temperature uniformity across 60 independent reaction chambers; however, by using larger chips and larger ITO substrate heaters, this number could easily be increased. Based on our observations with this current design, we recommend adding a 1 cm border between the chip/ITO edge and the area of the chip where amplification will take place.

Although the instrument presented here represents a promising platform for NAT in developing world settings, there remains a great deal of work before it can be applied as an effective diagnostic. In its present form, the µBAR does not provide sample preparation capabilities and requires that amplification reagents be mixed off-chip before sample loading. It also requires that DNA be supplied at fairly high concentrations (1000/uL, for example). Because of the small reaction volumes used in the microfluidic chambers, DNA purification and preconcentration will certainly be required for any clinical sample. Microfluidic technologies based on solid phase extraction using packed bead columns [15] or magnetic beads [25] are promising approaches which could be incorporated here. For blood-based diagnostics (such as HIV), plasma separation will also likely be required in order to minimize host genomic background. We recently demonstrated a microfluidic sedimentation technique, also based on the degas-driven flow, which effectively removes blood cells [14]. Aside from these sample preparation challenges, a method must be developed to lyophilize the LAMP reaction mix (enzymes, primers, dNTPs, etc.) into each reaction well before sealing the chip. Lyophilization would eliminate the need for a cold storage chain, and it would greatly streamline the diagnostic protocol. Including a unique set of primers in each reaction well would enable multiple genetic targets to be screened simultaneously. Finally, if the target is an RNA virus (such as HIV), reverse transcription can also be incorporated on-chip, or amplification chemistries which amplify RNA directly can be used such as nucleic acid sequence-based amplification, NASBA [26].

### Conclusions

In this paper, we have introduced the µBAR platform as a low-cost, portable alternative to traditional thermocyclers. The µBAR provides adequate fluorescence sensitivity and temperature uniformity for nucleic acid amplification assays. As a proof-of-principle, we have shown that the HIV *integrase* gene can be detected using the LAMP assay on the µBAR platform. The LAMP assay has been demonstrated for detecting drug resistance in TB from sputum samples [27] as well as detecting HIV [28] and malaria [29] in blood samples, and these assays can be readily adapted to the µBAR platform. However, much more work needs to be done to test this system on clinical samples and to develop on- or off-chip sample preparation protocols which can be run in resource-limited settings.

As applied to HIV, the µBAR platform would be ideal for screening high-risk populations in the developing world in order to immediately administer ART therapy without the need for a follow-up visit. Although other qualitative point-of-care tests currently exist for HIV, genetic information can provide more specific information about viral strain and drug susceptibility (e.g. HIV tropism), and quantitative viral load measurements can also be made with NAT. Drug susceptibility is also particularly important with Tuberculosis, where the spread of drug-resistant strains is a growing concern.

To help spur the development of low-cost point-of-care genetic diagnostic instruments, we have provided all of the designs, source code, and instructions necessary to build and run µBAR instruments. The supplemental information includes a full set of schematics (Figure S1 and Figure S2 in File S1), bill of materials and cost breakdown (Table S1 and Table S2 in File S1), microfluidic chip layout (Figure S3 in File S1), assembly instructions (Text S1 in File S1), and operating instructions (Text S2 in File S1). Additionally, the PCB design, firmware source code, enclosure CAD files, PC software source code, and microfluidic chip layout can be freely accessed in their original formats via GitHub (https://github.com/fbmyers/MicroBAR), and future revisions will be posted there. A summary of the files available on GitHub is provided in Text S3 in File S1. The µBAR hardware and firmware are based around the popular open-source Arduino platform, which enables rapid development and open collaboration.

## Supporting Information

File S1 **Figure S1,** Microcontroller Board Schematics and PCB Layout. **Figure S2,** Chip Reader Board Schematics and PCB Layout. **Table S1,** Bill of Materials and Cost Breakdown. **Table S2,** LAMP Assay Cost Estimates. **Figure S3,** Microfluidic Chip Layout. **Text S1,** Detailed Construction Procedure. **Text S2,** Operating Instructions. **Text S3,** Summary of files available in GitHub repository.
(PDF)

Movie S1 **Time lapse movie of degas driven sample loading in a microfluidic cartridge.** Initial loading is shown at 10X speed and once all chambers have begun filling, the video is sped up to 500X. The entire chip is fully loaded in about an hour.
(WMV)
