# Correction: A Handheld Point-of-Care Genomic Diagnostic System

**DOI:** 10.1371/annotation/902dc1bb-9ed7-4e91-a550-0f0a87ce17ba

**Published:** 2013-09-19

**Authors:** Frank B. Myers, Richard H. Henrikson, Jennifer M. Bone, Luke P. Lee

The name of the third author was given incorrectly. The correct name is: Jennifer M. Bone. 

The correct citation is: Myers FB, Henrikson RH, Bone JM, Lee LP (2013) A Handheld Point-of-Care Genomic Diagnostic System. PLoS ONE 8(8): e70266. doi:10.1371/journal.pone.0070266. 

The correct abbreviation in the Author Contributions statement is: JMB. 

