# Supplementary material for: A Handheld Point-of-Care Genomic Diagnostic System
Source: PLoS One. 2013 Aug 1;8(8):e70266. doi: 10.1371/journal.pone.0070266 (PMC3731356; doi:10.1371/journal.pone.0070266)
Supplement: File S1 — Figure S1, Microcontroller Board Schematics and PCB Layout. Figure S2, Chip Reader Board Schematics and PCB Layout. Table S1, Bill of Materials and Cost Breakdown. Table S2, LAMP Assay Cost Estimates. Figure S3, Microfluidic Chip Layout. Text S1, Detailed Construction Procedure. Text S2, Operating Instructions. Text S3, Summary of files available in GitHub repository. (PDF) [file pone.0070266.s001.pdf]

Figure S1 - Microcontroller Board Schematics and PCB Layout
a) Microcontroller and Peripheral Connectors

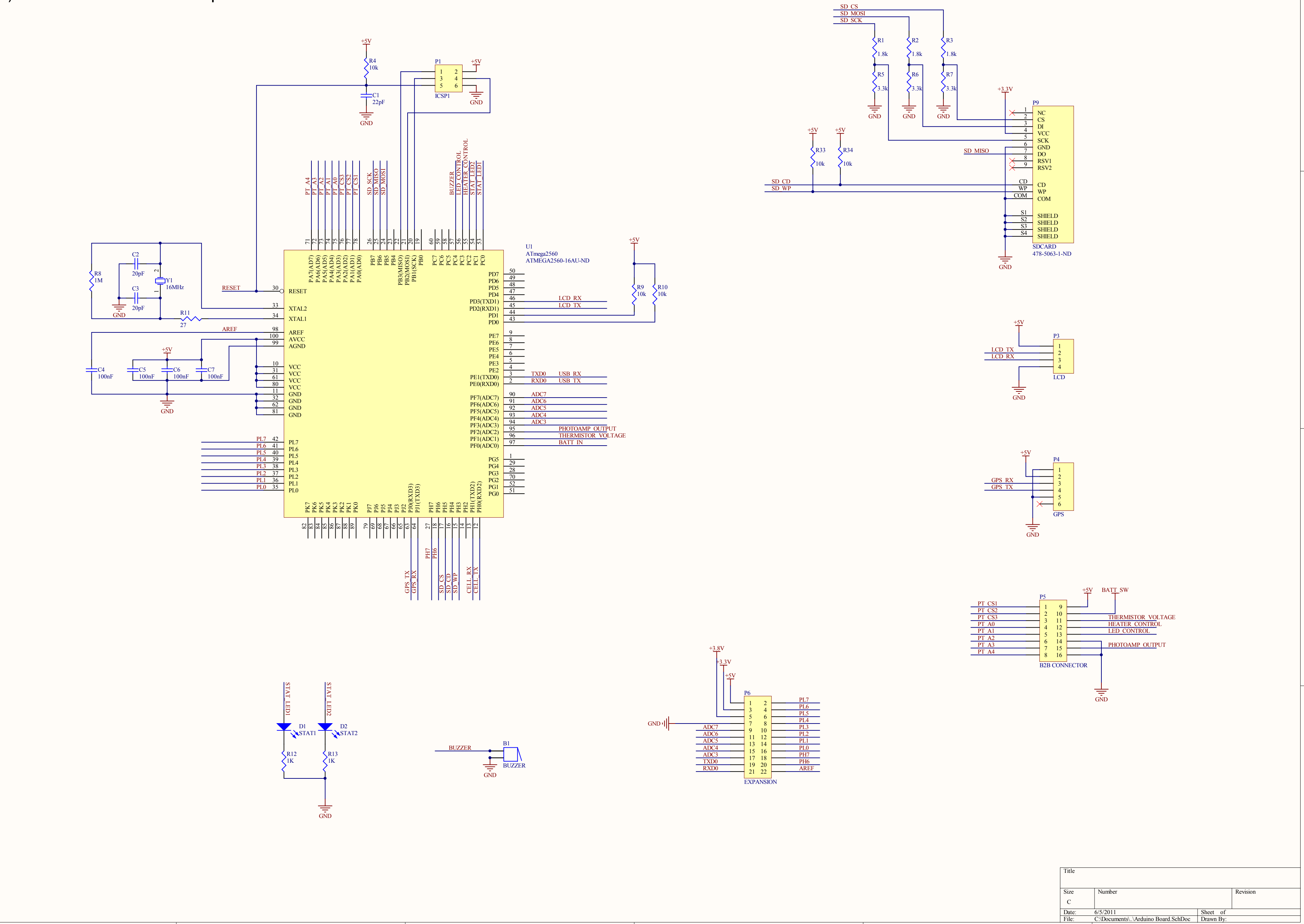

Figure S1 - Microcontroller Board Schematics and PCB Layout
b) Cell Phone Module

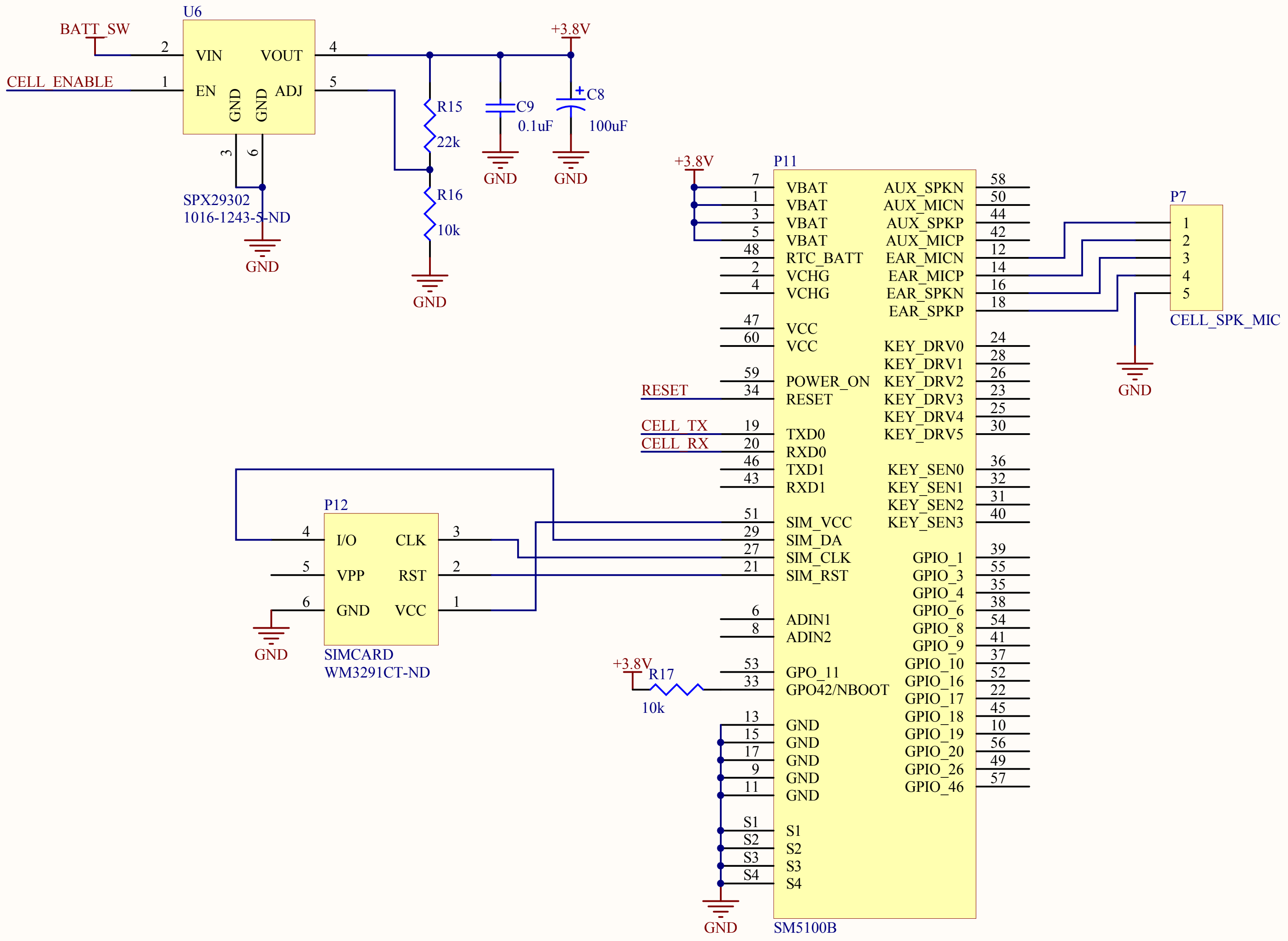

Title		
Size: A	Number	Revision
Date: 6/5/2011		Sheet of
File: C:\Documents\..\Cell Phone Module.SchDoc		Drawn By:

Figure S1 - Microcontroller Board Schematics and PCB Layout
c) Battery Charging and 5/3.3V Supply

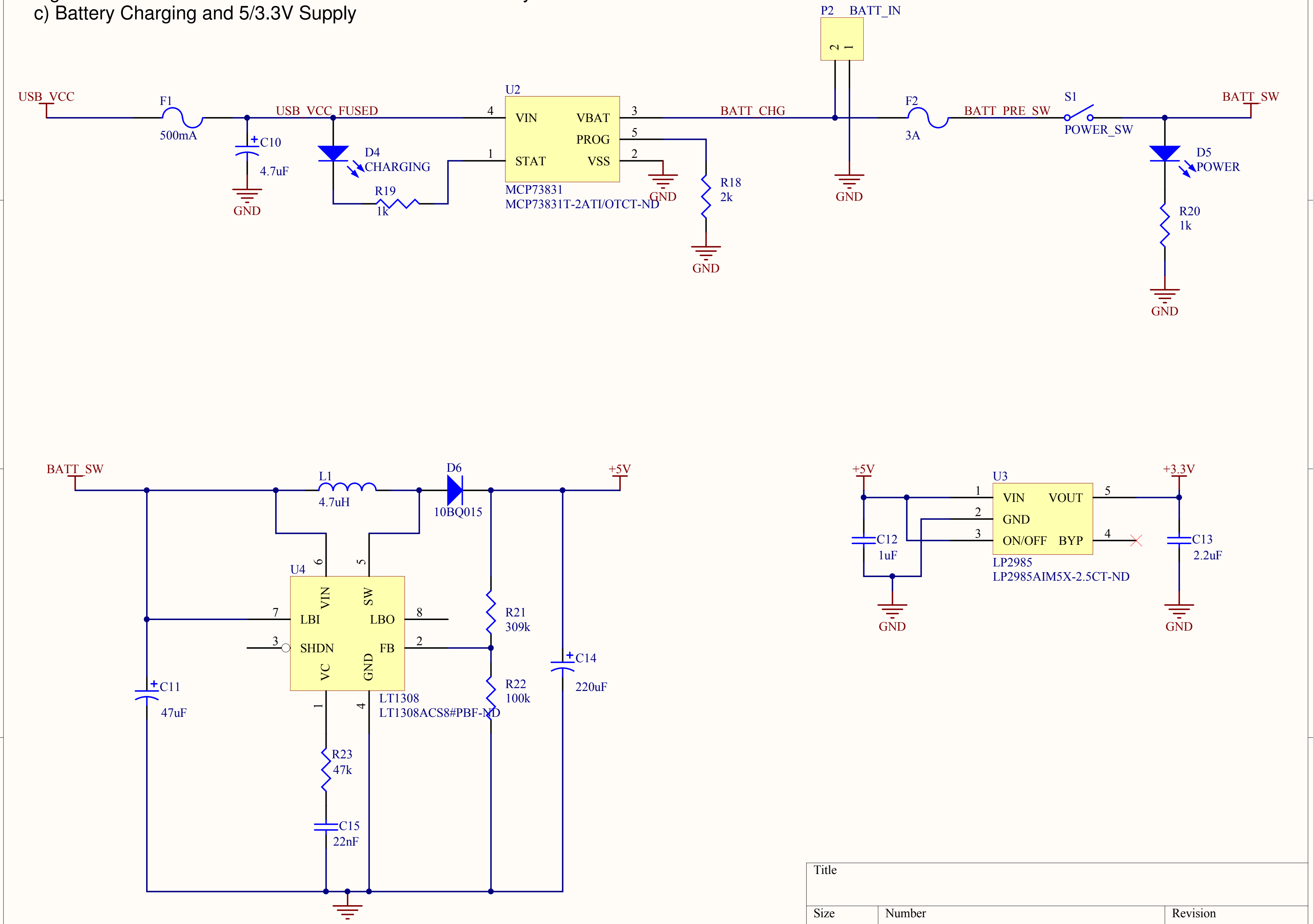

Title		
Size A	Number	Revision
Date: 6/5/2011		Sheet of
File: C:\Documents\..\Power Circuits.SchDoc		Drawn By:

Figure S1 - Microcontroller Board Schematics and PCB Layout
d) USB Interface

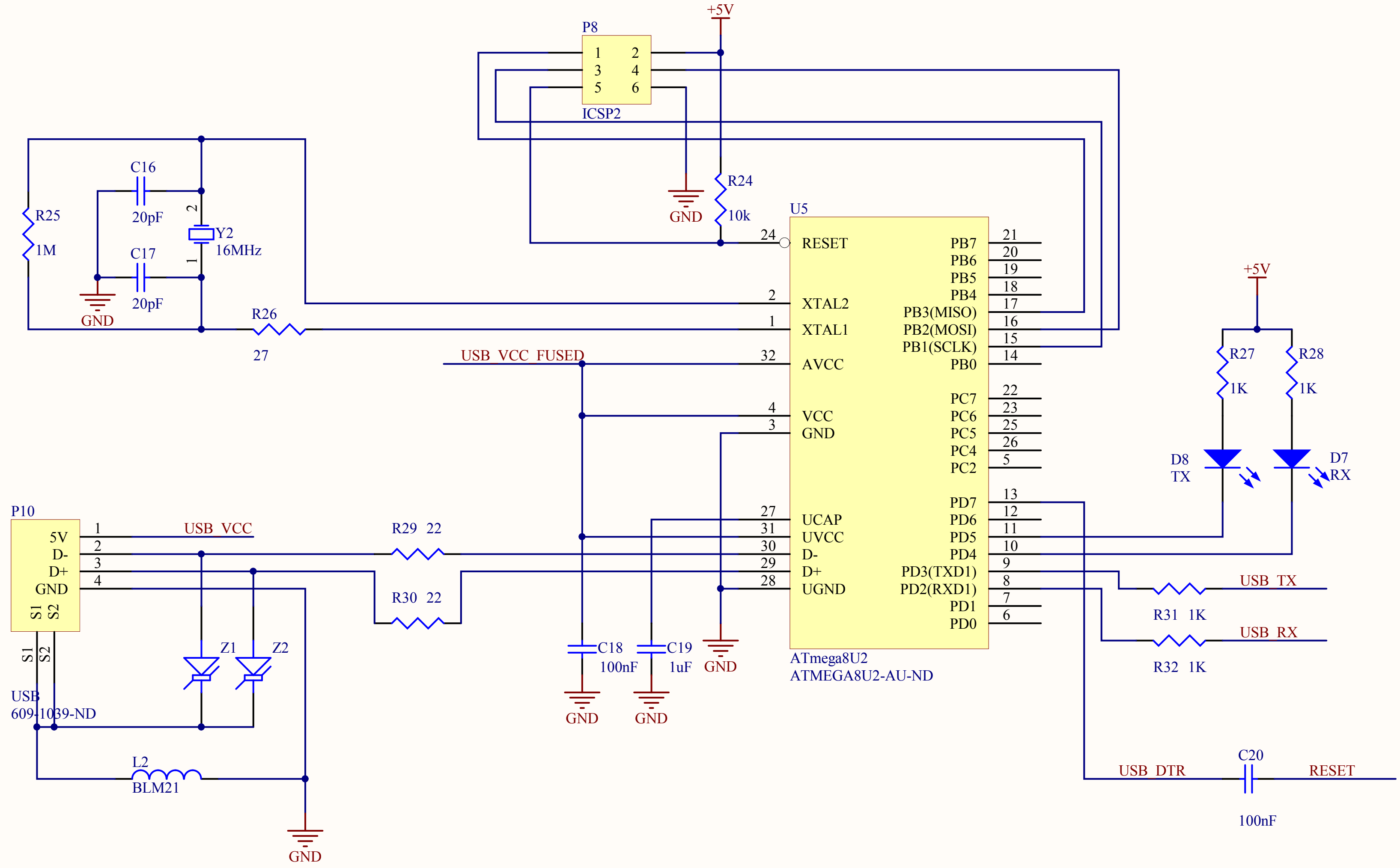

Title		
Size A	Number	Revision
Date: 6/5/2011		Sheet of
File: C:\Documents\..\USB Host.SchDoc		Drawn By:

Figure S1 - Microcontroller Board Schematics and PCB Layout
e) Board layout (2 layers, red=top, blue=bottom) with enclosure outline

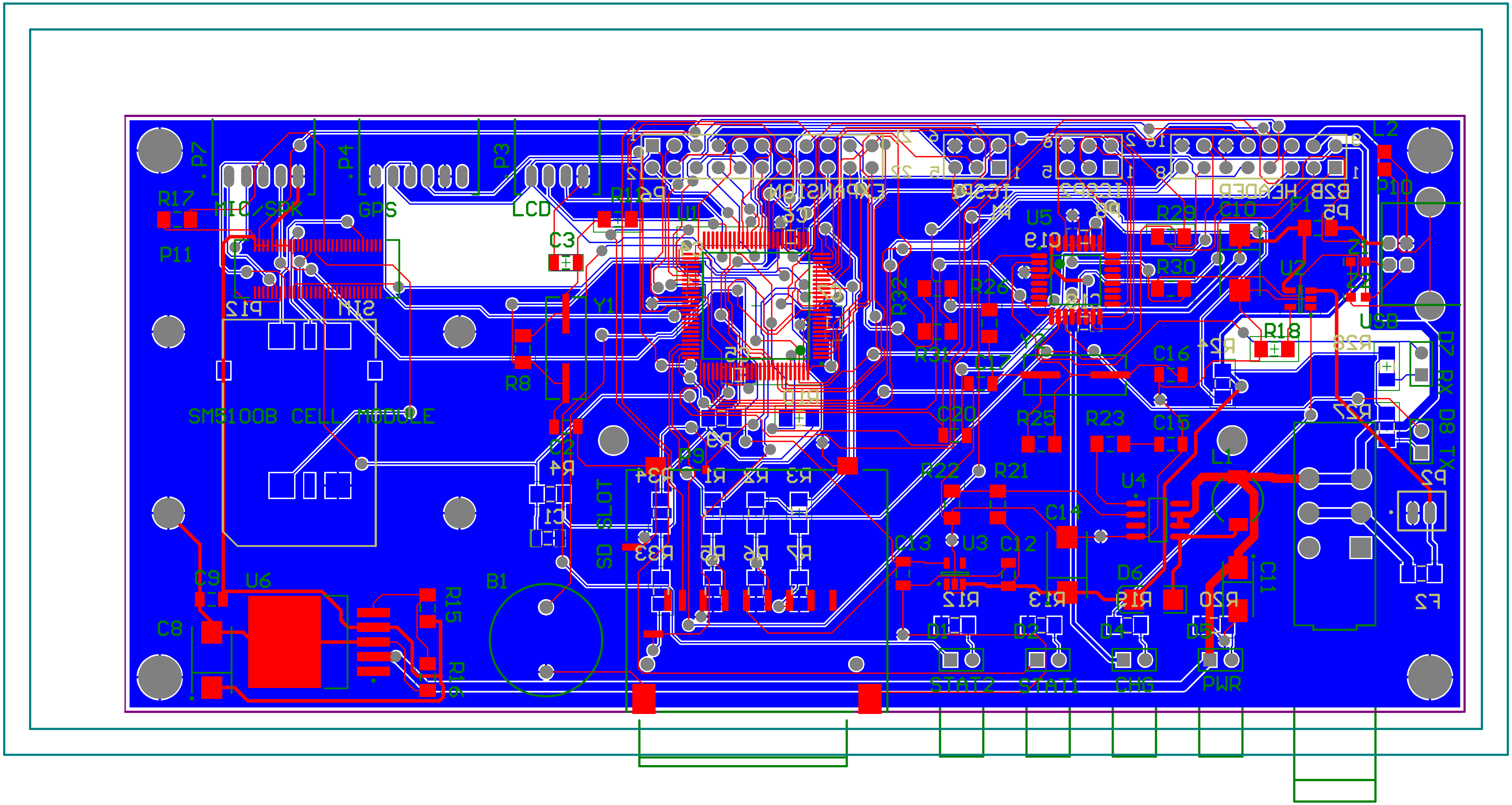

Figure S2 - Chip Reader Board Schematics and PCB Layout

a) Heater Controller

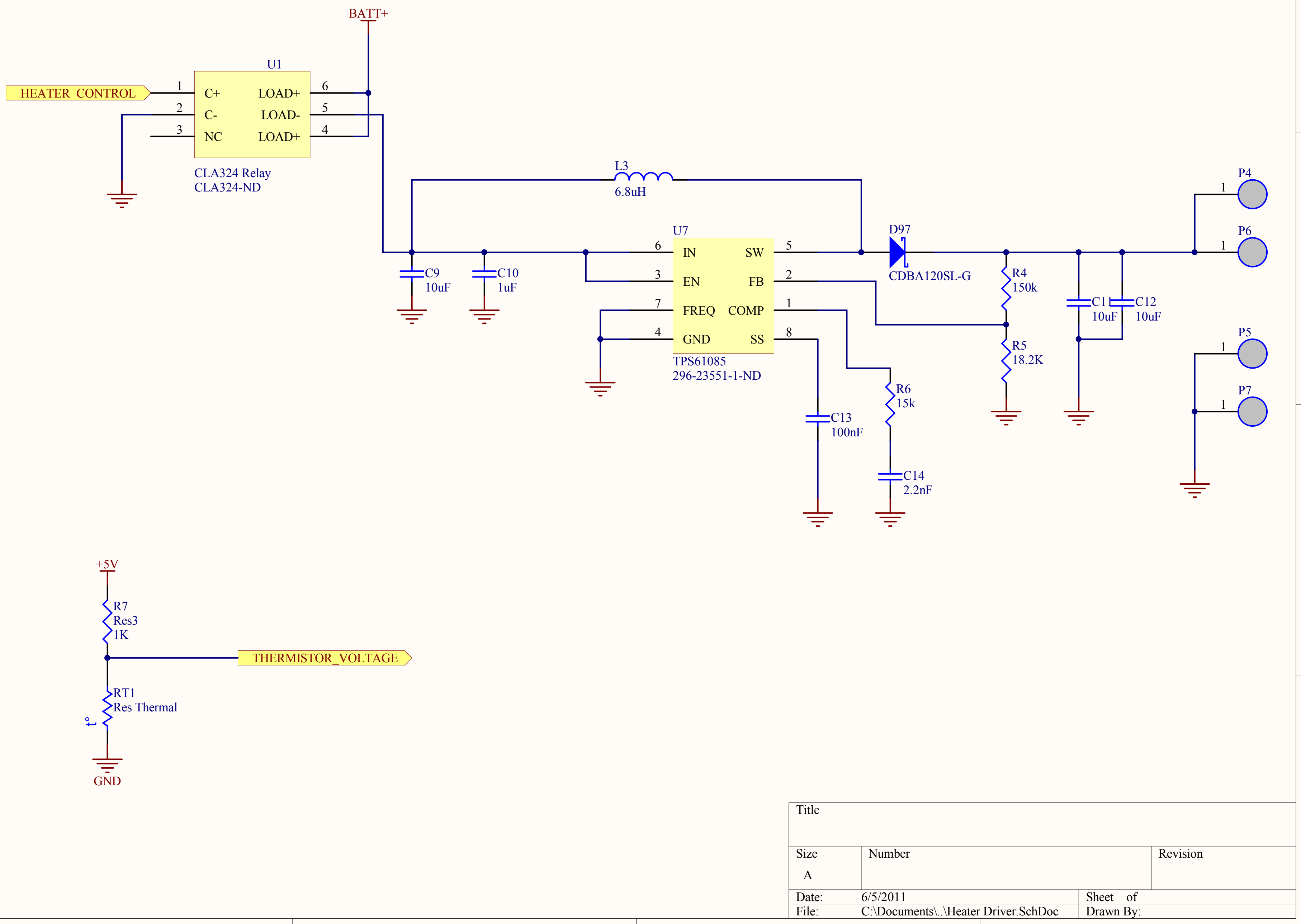

Figure S2 - Chip Reader Board Schematics and PCB Layout
b) LED Controller

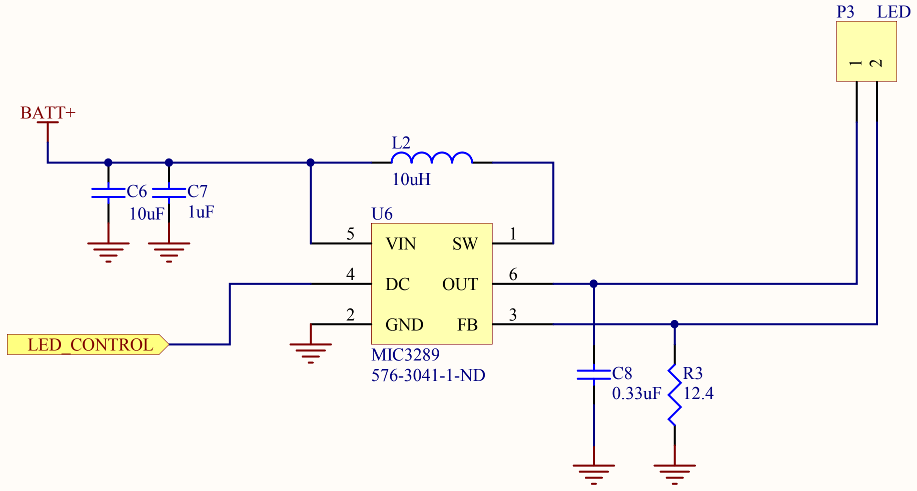

Title		
Size A	Number	Revision
Date: 6/5/2011		Sheet of
File: C:\Documents\..\LED Driver.SchDoc		Drawn By:

Figure S2 - Chip Reader Board Schematics and PCB Layout
c) Phototransistor Array and Photoamplifier

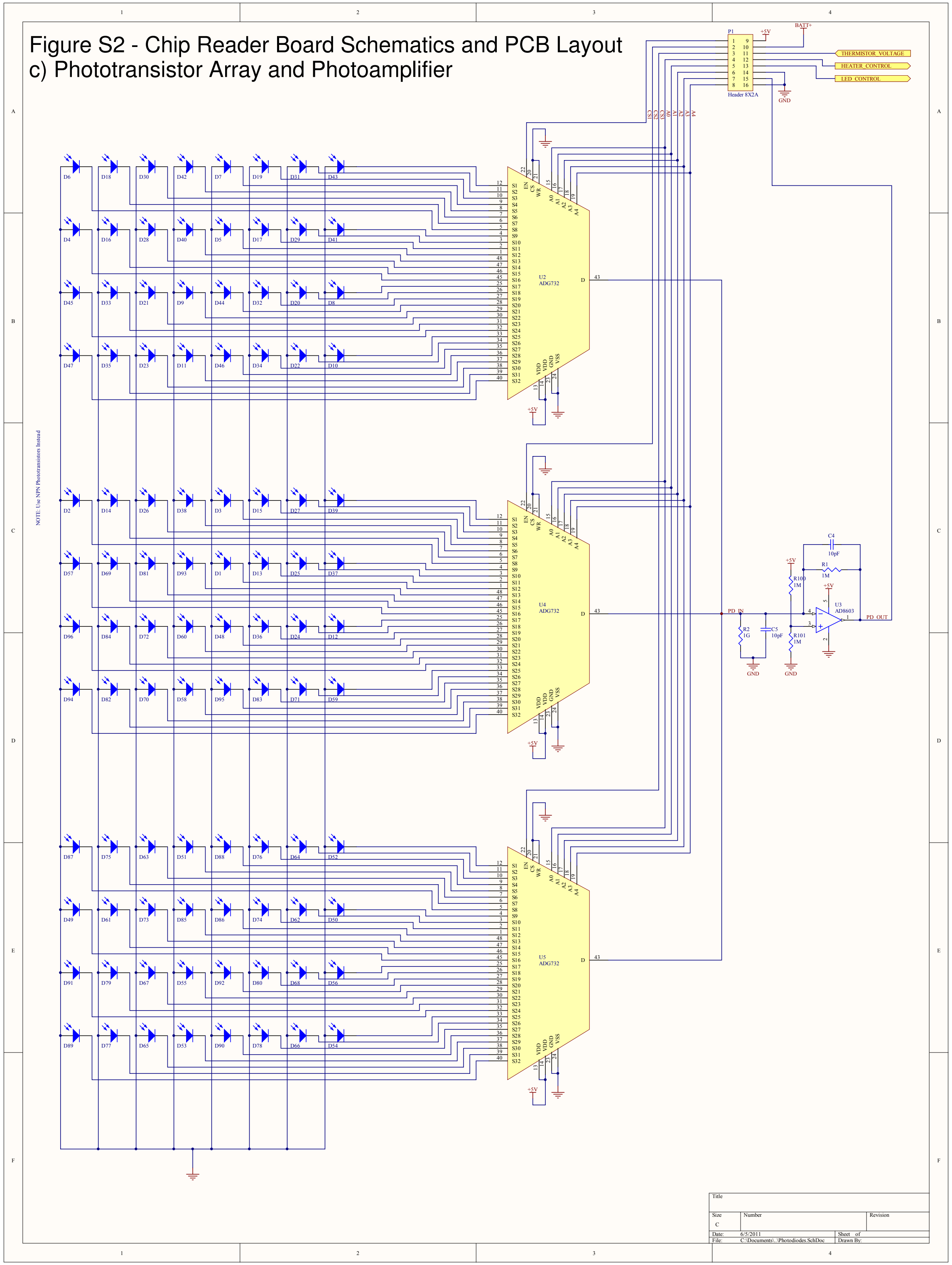

Figure S2 - Chip Reader Board Schematics and PCB Layout
d) Board layout (2 layers, red=top, blue=bottom) with enclosure outline

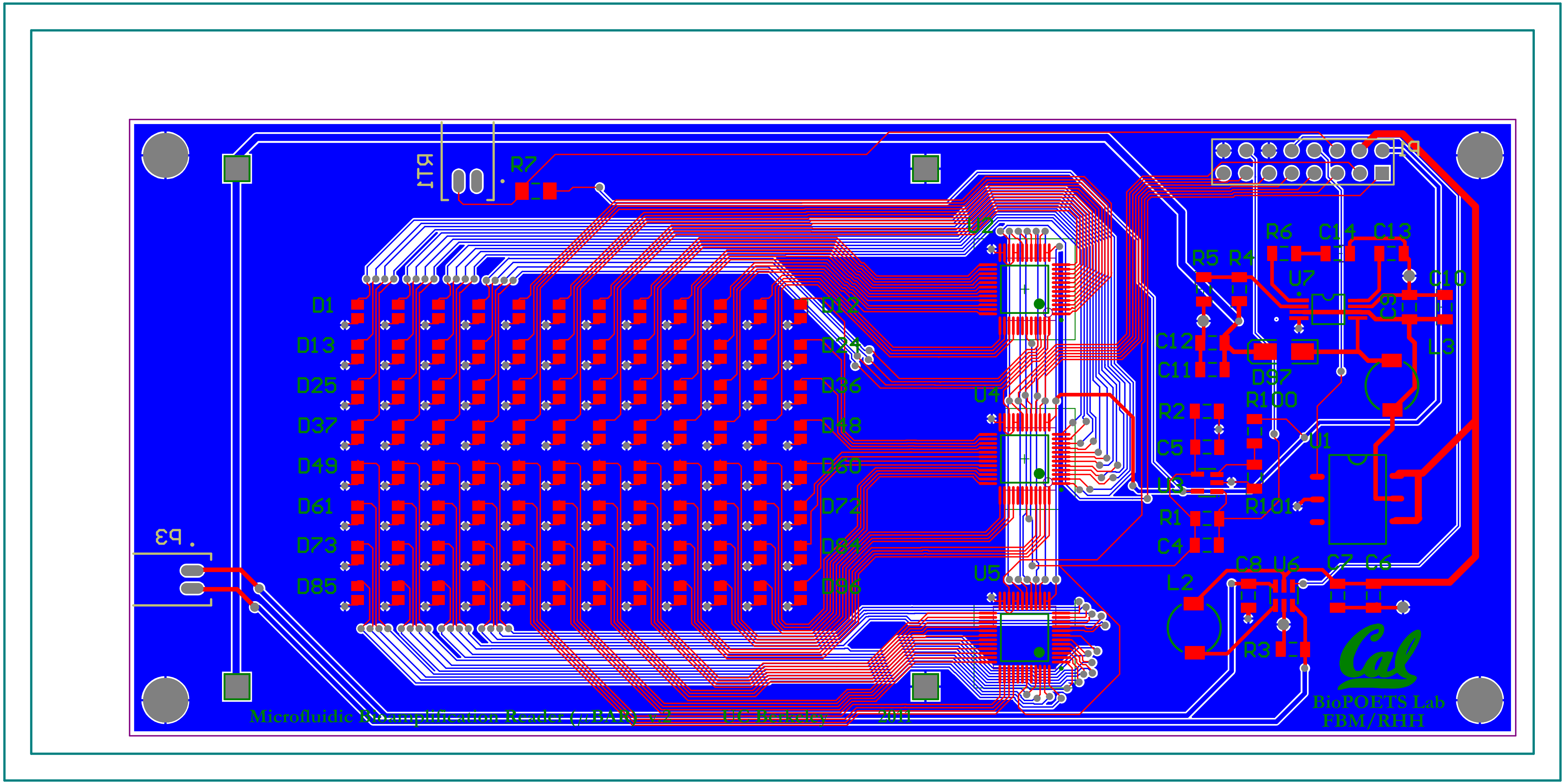

Table S1

MicroBAR v.2 Bill of Materials												
**System**	**Board**	**Designator**	**Qty**	**Vendor**	**Part #**	**Mfg #**	**Description**	**Function**	**Unit Price (proto)**	**Unit Price (>5000)**	**Ext Price (proto)**	**Ext price (>5000)**
**ITO Heater**	Top	U1	1	Digikey	CLA324-ND	LCA717S	RELAY OPTOMOS 2A SPST-NO 6-SMD	Heater Relay	6.18	2.575	6.18	2.575
**ITO Heater**	Top	U7	1	Digikey	296-23551-1-ND	TPS61085PWR	IC STEP-UP DC-DC CONV 8-TSSOP	Heater boost converter	3.33	1.283	3.33	1.283
**ITO Heater**	Top	C9,C11,C12	3	Digikey			10uF (1206)	input/output filter caps	0.15	0.02	0.45	0.06
**ITO Heater**	Top	C10	1	Digikey			1uF (1206)	output filter cap	0.15	0.02	0.15	0.02
**ITO Heater**	Top	C13	1	Digikey			100nF (1206)		0.15	0.02	0.15	0.02
**ITO Heater**	Top	C14	1	Digikey			2.2nF (1206)		0.15	0.02	0.15	0.02
**ITO Heater**	Top	R4	1	Digikey			150k (1206)		0.05	0.01	0.05	0.01
**ITO Heater**	Top	R5	1	Digikey			18.2k (1206)		0.05	0.01	0.05	0.01
**ITO Heater**	Top	R6	1	Digikey			15k (1206)		0.05	0.01	0.05	0.01
**ITO Heater**	Top	R7	1	Digikey			1k (1206)	Half bridge for thermistor	0.05	0.01	0.05	0.01
**ITO Heater**	Top	L3	1	Digikey	490-5270-1-ND	LQH55PN6R8NR(	INDUCTOR POWER 6.8UH 1450MA 2220	6.8uH heater inductor	0.44	0.172	0.44	0.172
**ITO Heater**	Top	D97	1	Digikey	641-1255-1-ND	CDBA120SL-G	DIODE SCHOTTKY 1A 20V SMA	heater diode	0.82	0.209	0.82	0.209
**ITO Heater**	Top	RT1	1	Digikey	615-1043-ND	PPG102A1RD	DETECTOR RTD TF 1K OHM +/-0.06%	Thermistor	18.5	2.06	18.5	2.06
**ITO Heater**	Top	RT1	1	Digikey	455-1719-ND	S2B-PH-K-S(LF)(S	CONN HEADER PH SIDE 2POS 2MM	header for thermistor	0.1	0.047	0.1	0.047
**ITO Heater**	Top	RT1	1	Digikey	455-1165-ND	PHR-2	CONN HOUSING PH 2POS 2MM WHITE	2-pin housing for thermistor	0.05	0.024	0.05	0.024
**ITO Heater**	Top	RT1	2	Digikey	455-2148-1-ND	SPH-002T-P0.5L	CONN TERMINAL 28-24AWG TIN	JST PH pins	0.027	0.013	0.054	0.026
**ITO Heater**	Top	RT1	1	Digikey	MC06G-300-ND	3365/06 300SF	CABLE 6 COND 300FT FLAT GRAY	2-conductor ribbon cable (3 inches)	0.01	0.01	0.01	0.01
**ITO Heater**	Top		1	Delta Tech	CB-50IN-S205	CB-50IN-S205	5-15 ohm, 50x75x0.5mm ITO slides	Heater	20	5	20	5
**ITO Heater**	Top		1	Ace HW			Brass Bar Stock	Heater Contacts	0.5	0.25	0.5	0.25
**ITO Heater**	Top		1	Fry's			Electrically-conductive Epoxy	Heater Contacts	0.2	0.1	0.2	0.1
**ITO Heater**	Top		1	Ace HW			Thermally-conductive Epoxy	Glue for Thermistor	0.05		0.05	0
										**TOTAL:**	**51.334**	**11.916**
**LED Driver**	Top	U6	1	Digikey	576-3041-1-ND	MIC3289-24YD6	IC LED DRIVR WHITE BCKLGT TSOT-6	LED booster	1.9	0.973	1.9	0.973
**LED Driver**	Top	C6	1	Digikey			10uF (1206)		0.15	0.02	0.15	0.02
**LED Driver**	Top	C7	1	Digikey			1uF (1206)		0.15	0.02	0.15	0.02
**LED Driver**	Top	C8	1	Digikey	478-6028-1-ND	06033D334KAT2	CAP CER .33UF 25V X5R 0603 (sub w/ 1206)	0.33uF	0.45	0.126	0.45	0.126
**LED Driver**	Top	R3	1	Digikey	P12.4CCT-ND	ERJ-6ENF12R4V	RES 12.4 OHM 1/8W 1% 0805 SMD	current sense resistor	0.07	0.005	0.07	0.005
**LED Driver**	Top	L2	1	Digikey	490-5271-1-ND	LQH55PN100MR	INDUCTOR POWER 10UH 1250MA 2220	10uH LED inductor	0.44	0.172	0.44	0.172
**LED Driver**	Top	P3	1	Digikey	455-1719-ND	S2B-PH-K-S(LF)(S	CONN HEADER PH SIDE 2POS 2MM	header for leds	0.1	0.047	0.1	0.047
**LED Driver**	Top	P3	1	Digikey	455-1165-ND	PHR-2	CONN HOUSING PH 2POS 2MM WHITE	2-pin housing for leds	0.05	0.024	0.05	0.024
**LED Driver**	Top	P3	2	Digikey	455-2148-1-ND	SPH-002T-P0.5L	CONN TERMINAL 28-24AWG TIN	JST PH pins	0.027	0.013	0.054	0.026
**LED Driver**	Top	P3	1	Digikey	MC06G-300-ND	3365/06 300SF	CABLE 6 COND 300FT FLAT GRAY	2-conductor ribbon cable (3 inches)	0.01	0.01	0.01	0.01
**LED Driver**	Top	P3	3	Digikey	P14150CT-ND	LNJ923W8CRA1	LED BLUE SIDE VIEW SMD	Blue LEDs	0.57	0.229	1.71	0.687
**LED Driver**	Top		1	Ace HW			Liquid Electrical Tape	Optical shielding	0.05	0.02	0.05	0.02
										**TOTAL:**	**5.134**	**2.13**
**Phototransistor Array**	Top	D1-D96	96	Digikey	475-1268-1-ND	SFH 3710-3/4-Z	PHOTOTRANSISTOR ALS SMD CHIPLED	Phototransistors	0.563	0.483	54.048	46.368
**Phototransistor Array**	Top	U2,U4,U5	3	Digikey	ADG732BSUZ-ND	ADG732BSUZ	IC MULTIPLEXER 32X1 48TQFP	MUXes	12.13	6.062	36.39	18.186
**Phototransistor Array**	Top	U3	1	Digikey	AD8603AUJZREEL7CT-N	AD8603AUJZ-REE	IC OPAMP GP R-R CMOS TSOT23-5	transimpedance opamp	2.07	0.797	2.07	0.797
**Phototransistor Array**	Top	C4	1	Digikey			10pF	fb capacitor	0.15	0.02	0.15	0.02
**Phototransistor Array**	Top	C5	1	Digikey			10pF - not necessary?	input capacitor	0	0	0	0
**Phototransistor Array**	Top	R1	1	Digikey			1M	fb resistor	0.1	0.01	0.1	0.01
**Phototransistor Array**	Top	R2	1	Digikey			1G - not necessary?	input resistor	0	0	0	0
**Phototransistor Array**	Top	R100-101	2	Digikey			1M	bias resistors	0.1	0.01	0.2	0.02
**Phototransistor Array**	Top		1	McMaster	8647K108		Neoprene/EPDM/SBR, Soft, 1/16" Thick, 42" Width	black foam	0.04	0.04	0.04	0.04
**Phototransistor Array**	Top		1	McMaster	7769T88 (#89)		Light Filters 20" X 24" Sheet	"Moss Green" gel #89	0.16	0.16	0.16	0.16
										**TOTAL:**	**93.158**	**65.601**
**Microcontroller**	Bot	U1	1	Digikey	ATMEGA2560-16AU-NI	ATMEGA2560-16	IC AVR MCU 256K 16MHZ 100TQFP	microcontroller	17.97	11.251	17.97	11.251
**Microcontroller**	Bot	C1	1	Digikey			22pF (1206)	cap on reset line	0.15	0.02	0.15	0.02
**Microcontroller**	Bot	C2-3	2	Digikey	478-3735-1-ND	08051A200JAT2A	CAP CERM 20PF 5% 100V NP0 0805	xtal caps	0.23	0.038	0.46	0.076
**Microcontroller**	Bot	C4-7	4	Digikey			100nF (1206)	decoupling caps	0.15	0.02	0.6	0.08
**Microcontroller**	Bot	R4	1	Digikey			10k (1206)	res on reset line	0.1	0.01	0.1	0.01
**Microcontroller**	Bot	R8	1	Digikey			1M (1206)	res on xtal	0.1	0.01	0.1	0.01
**Microcontroller**	Bot	R11	1	Digikey			27 ohm (1206)	res on xtal	0.1	0.01	0.1	0.01
**Microcontroller**	Bot	Y1	1	Digikey	XC1282CT-ND	ECS-160-20-5PX-	CRYSTAL 16.00MHZ 20PF SMD	xtal	0.56	0.25	0.56	0.25
**Microcontroller**	Bot	R9-10	2	Digikey			10k (1206)	pullups on PD1/PD0	0.1	0.01	0.2	0.02
**Microcontroller**	Bot	P1	1	Digikey	609-3218-ND	67996-406HLF	CONN HEADER 6POS .100 STR TIN	ICSP1 header	0.43	0	0.43	0
										**TOTAL:**	**20.67**	**11.727**
**USB Interface**	Bot	U5	1	Digikey	ATMEGA8U2-AU-ND	ATMega8U2-AU	MCU AVR 8K FLASH 16MHZ 32TQFP	usb controller	3.7	2.352	3.7	2.352
**USB Interface**	Bot	C16-17	2	Digikey	478-3735-1-ND	08051A200JAT2A	CAP CERM 20PF 5% 100V NP0 0805	xtal caps	0.23	0.038	0.46	0.076
**USB Interface**	Bot	R25	1	Digikey			1M (1206)	res on xtal	0.1	0.01	0.1	0.01
**USB Interface**	Bot	R26	1	Digikey			27 ohm (1206)	res on xtal	0.1	0.01	0.1	0.01
**USB Interface**	Bot	Y2	1	Digikey	XC1282CT-ND	ECS-160-20-5PX-	CRYSTAL 16.00MHZ 20PF SMD	xtal	0.56	0.25	0.56	0.25
**USB Interface**	Bot	R24	1	Digikey			10k (1206)	pullup on reset line	0.1	0.01	0.1	0.01
**USB Interface**	Bot	R29-30	2	Digikey			22 ohm (1206)	USB data line resistors	0.1	0.01	0.2	0.02
**USB Interface**	Bot	Z1-2	2	Digikey	F2594CT-ND	PGB1010603MR	SUPPRESSOR ESD 24VDC 0603 SMD	USB data line ESD protection	0.74	0.196	1.48	0.392
**USB Interface**	Bot	L2	1	Digikey	490-1038-1-ND	BLM21AG121SN	FERRITE CHIP 120 OHM 200MA 0805	ESD protection inductor	0.33	0.039	0.33	0.039
**USB Interface**	Bot	P10	1	Digikey	609-1039-ND	61729-0010BLF	CONN RCPT USB TYPE B R/A PCB	USB-B port	1.29	0.474	1.29	0.474
**USB Interface**	Bot	C18	1	Digikey			100nF (1206)		0.15	0.01	0.15	0.01
**USB Interface**	Bot	C19	1	Digikey			1uF (1206)		0.15	0.01	0.15	0.01
**USB Interface**	Bot	R27-28	2	Digikey			1k (1206)	TX/RX pullups	0.1	0	0.2	0
**USB Interface**	Bot	D7-8	2	Digikey	67-1046-ND	SSL-LX2573GD	LED 2X5MM RECTANGULAR GREEN DIFF	TX/RX LEDs	0.37	0	0.74	0
**USB Interface**	Bot	R31-32	2	Digikey			1k (1206)	data line resistors	0.1	0.01	0.2	0.02
**USB Interface**	Bot	C20	1	Digikey			100nF (1206)	reset DTR line cap	0.15	0.02	0.15	0.02
**USB Interface**	Bot	P8	1	Digikey	609-3218-ND	67996-406HLF	CONN HEADER 6POS .100 STR TIN	ICSP2 header	0.43	0	0.43	0
**USB Interface**			1	Digikey	Q361-ND	3021001-03	CBL USB A-B CON 3' 28/28 AWG	3' USB cable	3.15	1.23	3.15	1.23
										**TOTAL:**	**13.49**	**4.923**
**SD Card Reader**	Bot	P9	1	Digikey	478-5063-1-ND	14573800930285	CONNECTOR SD CARD W/BOSS	SD card slot	4.7	2.916	4.7	2.916
**SD Card Reader**	Bot	R33-34	2	Digikey			10k (1206)	card detect / write protect pullups	0.1	0.01	0.2	0.02
**SD Card Reader**	Bot	R1-3	3	Digikey			1.8k (1206)	SD level shifter res	0.1	0.01	0.3	0.03
**SD Card Reader**	Bot	R5-7	3	Digikey			3.3k (1206)	SD level shifter res	0.1	0.01	0.3	0.03
**SD Card Reader**	Bot	U3	1	Digikey	296-18479-1-ND	LP2985A-33DBVI	IC LDO REG 3.3V 150MA SOT23-5	3.3V regulator	0.84	0.276	0.84	0.276
**SD Card Reader**	Bot	C12	1	Digikey			1uF (1206)	input cap for 3.3v reg	0.15	0.02	0.15	0.02
**SD Card Reader**	Bot	C13	1	Digikey			2.2uF(1206)	output cap for 3.3v reg	0.15	0.02	0.15	0.02
**SD Card Reader**	Bot		1	Amazon				512MB SD Card	6	3	6	3
										**TOTAL:**	**12.64**	**6.312**
**Cell Module**	Bot	P11	1	Digikey	255-2997-1-ND	AXK5F60547YG	CONN SOCKET BRD/BRD .5MM 60POS	60-pin socket for cell module	6.79	3.514	6.79	3.514
**Cell Module**	Bot	P12	1	Digikey	WM3291CT-ND	473880001	1.90MM SIM CARD READER CONN	SIM card socket	1.51	0.986	1.51	0.986
**Cell Module**	Bot	U6	1	Digikey	1016-1243-5-ND	SPX29302T5-L	IC REG LDO ADJ 3A TO263-5	3.8V 3A regulator	2.68	1.123	2.68	1.123
**Cell Module**	Bot	R15	1	Digikey			22k	3.8V voltage set res	0.1	0.01	0.1	0.01
**Cell Module**	Bot	R16	1	Digikey			10k	3.8V voltage set res	0.1	0.01	0.1	0.01
**Cell Module**	Bot	C8	1	Digikey	478-1777-1-ND	TPSD107K010R0	CAP TANT LOESR 100UF 10V 10% SMD	output cap for 3.8V reg	1.13	0.36	1.13	0.36
**Cell Module**	Bot	C9	1	Digikey			0.1uF	output cap for 3.8V reg	0.15	0.02	0.15	0.02
**Cell Module**	Bot	R17	1	Digikey			10k	pullup for boot res	0.1	0.01	0.1	0.01
**Cell Module**	Bot	P7	1	Digikey	455-1722-ND	S5B-PH-K-S(LF)(S	CONN HEADER PH SIDE 5POS 2MM	5-pin JST PH header for cell phone spkr/mic	0.2	0	0.2	0
**Cell Module**	Bot		1	Digikey	455-1163-ND	PHR-5	CONN HOUSING PH 5POS 2MM WHITE	5-pin housing - for mic/spkr cable	0.07	0	0.07	0
**Cell Module**	Bot		1	Sparkfun	CEL-09533		GSM/GPRS Module - SM5100B	SM5100B Cell Module	59.95	47.96	59.95	47.96
**Cell Module**	Bot		1	Sparkfun	CEL-00675		Quad-band Cellular Duck Antenna SMA	Cell Antenna	7.95	6.39	7.95	6.39
**Cell Module**	Bot		1					Prepay SIM card	0	0	0	0
										**TOTAL:**	**80.73**	**60.383**
**GPS Module**	Bot	P4	1	Digikey	455-1721-ND	S4B-PH-K-S(LF)(S	CONN HEADER PH SIDE 4POS 2MM	6-pin JST header for GPS	0.17	0.076	0.17	0.076
**GPS Module**	Bot	P4	1	Digikey	455-1396-ND	SHR-06V-S	CONN HOUSING SH 6POS 1MM WHITE	housing for GPS connector	0.11	0.049	0.11	0.049
**GPS Module**	Bot	P4	6	Digikey	455-2148-1-ND	SPH-002T-P0.5L	CONN TERMINAL 28-24AWG TIN	JST PH pins	0.027	0.013	0.162	0.078
**GPS Module**	Bot	P4	1	Digikey	MC06G-300-ND	3365/06 300SF	CABLE 6 COND 300FT FLAT GRAY	6-cond 28 awg ribbon (3 inches)	0.03	0.03	0.03	0.03
**GPS Module**	Bot	P4	1	Digikey	455-1396-ND	SHR-06V-S	CONN HOUSING SH 6POS 1MM WHITE	6-pin JST housing - GPS end	0.11	0.049	0.11	0.049
**GPS Module**	Bot	P4	1	Digikey	455-1561-1-ND	SSH-003T-P0.2	CONN TERM SH CRIMP 28-32AWG TIN	JST SH pins	0.049	0.032	0.049	0.032
**GPS Module**			1	Sparkfun	GPS-00465		20 Channel EM-406A SiRF III Receiver with Antenna	GPS module	59.95	47.96	59.95	47.96
										**TOTAL:**	**60.581**	**48.274**
**5V Power Supply**	Bot	U4	1	Digikey	LT1308ACS8#PBF-ND	LT1308ACS8#PBF	IC DC/DC CONV SINGLE CELL 8-SOIC	5V, 1A regulator	6.5	3.634	6.5	3.634
**5V Power Supply**	Bot	C11	1	Digikey	478-3911-1-ND	TAJC476M010RN	CAP TANTALUM 47UF 10V 20% SMD	5V booster input cap	0.46	0.147	0.46	0.147
**5V Power Supply**	Bot	C15	1	Digikey			22nF (1206)		0.15	0.02	0.15	0.02
**5V Power Supply**	Bot	C14	1	Digikey	478-3061-1-ND	TPSD227M006R(	CAP TANT LOESR 220UF 6.3V 20%SMD	5V booster output cap	3.5	1.12	3.5	1.12
**5V Power Supply**	Bot	R23	1	Digikey			47k (1206)		0.1	0.01	0.1	0.01
**5V Power Supply**	Bot	R21	1	Digikey			309k (1206)		0.1	0.01	0.1	0.01
**5V Power Supply**	Bot	R22	1	Digikey			100k (1206)		0.1	0.01	0.1	0.01
**5V Power Supply**	Bot	L1	1	Digikey	490-5269-1-ND	LQH55PN4R7NR(	INDUCTOR POWER 4.7UH 1750MA 2220	5V booster inductor	0.44	0.172	0.44	0.172
**5V Power Supply**	Bot	D6	1	Digikey	10BQ015PBFCT-ND	10BQ015TRPBF	DIODE SCHOTTKY 15V 1A SMB	5V booster diode	1.02	0.336	1.02	0.336
										**TOTAL:**	**12.37**	**5.459**
**LiPo Battery/USB Charger**	Bot	U2	1	Digikey	MCP73831T-2ATI/OTCT	MCP73831T-2AT	IC CONTROLLR LI-ION 4.2V SOT23-5	lipo charger	0.68	0.42	0.68	0.42
**LiPo Battery/USB Charger**	Bot	D4	1	Digikey	67-1050-ND	SSL-LX2573YD	LED 2X5MM RECTANGULAR YEL DIFF	charging LED (yellow)	0.28	0.08	0.28	0.08
**LiPo Battery/USB Charger**	Bot	R19	1	Digikey			1k (1206)	charging LED res	0.1	0.01	0.1	0.01
**LiPo Battery/USB Charger**	Bot	R18	1	Digikey			2k (1206)	current selector for charging IC	0.1	0.01	0.1	0.01
**LiPo Battery/USB Charger**	Bot	F1	1	Digikey	507-1075-1-ND	C1Q 500	FUSE 500MA 125V 1206 FAST C1Q	500mA USB charging fuse	0.55	0.142	0.55	0.142
**LiPo Battery/USB Charger**	Bot	F2	1	Digikey	507-1082-1-ND	C1Q 3	3A fuse	3A battery fuse	0.55	0.142	0.55	0.142
**LiPo Battery/USB Charger**	Bot	C10	1	Digikey	478-3934-1-ND	TAJD475M035RN	CAP TANTALUM 4.7UF 35V 20% SMD	lipo charger cap	0.97	0.35	0.97	0.35
**LiPo Battery/USB Charger**	Bot	P2	1	Digikey	455-1704-ND	B2B-PH-K-S(LF)(S	CONN HEADER PH TOP 2POS 2MM	battery connector	0.1	0.0476	0.1	0.0476
**LiPo Battery/USB Charger**	Bot	P2	1	Sparkfun	PRT-08484		6Ah LiPo battery w/ JST connector	battery (many options for this)	39.95	31.96	39.95	31.96
**LiPo Battery/USB Charger**	Bot		2					screws/brackets for mounting battery	0.1	0.1	0.2	0.2
										**TOTAL:**	**43.48**	**33.3616**
**Touchscreen LCD**		P3	1	Digikey	455-1721-ND	S4B-PH-K-S(LF)(S	CONN HEADER PH SIDE 4POS 2MM	header for LCD	0.17	0.076	0.17	0.076
**Touchscreen LCD**		P3	1	Digikey	455-1164-ND	PHR-4	CONN HOUSING PH 4POS 2MM WHITE	4-pin housing	0.07	0.03	0.07	0.03
**Touchscreen LCD**		P3	4	Digikey	455-2148-1-ND	SPH-002T-P0.5L	CONN TERMINAL 28-24AWG TIN	JST PH pins	0.027	0.013	0.108	0.052
**Touchscreen LCD**		P3	1	Digikey	MC06G-300-ND	3365/06 300SF	CABLE 6 COND 300FT FLAT GRAY	4-cond 28 awg ribbon	0.02	0.02	0.02	0.02
**Touchscreen LCD**			1	Digikey	681-1018-ND	MK-480272C	KIT COLOR MODULE 480X272 TFT	4.3" Color LCD Touchscreen	223.44	50	223.44	50
										**TOTAL:**	**223.808**	**50.178**
**General**	Bot	D1,D2	2	Digikey	67-1047-ND	SSL-LX2573ID	LED 2X5MM RECTANGULAR RED DIFF	Status LEDs (red)	0.37	0.079	0.74	0.158
**General**	Bot	D5	1	Digikey	67-1046-ND	SSL-LX2573GD	LED 2X5MM RECTANGULAR GREEN DIFF	power LED (grn)	0.37	0.074	0.37	0.074
**General**	Bot	R12,R13,R20	3	Digikey			1k (1206)	Resistors for status/power LEDs	0.1	0.01	0.3	0.03
**General**	Bot	S1	1	Digikey	401-1223-ND	F2UEE	SWITCH PB DPDT ALT ACT SILVER	power switch	2.32	1.036	2.32	1.036
**General**	Bot	S1	1	Digikey	401-1221-ND	F1204	SW CAP RECT GREY 5.5MM ACT	power switch gray cap	1.05	0.355	1.05	0.355
**General**	Bot	B1	1	Digikey	458-1066-ND	PK-12N40PQ	BUZZER PIEZO 4.1KHZ 14MM PC MT	Buzzer	2.43	1.978	2.43	1.978
**General**	Bot	P6	1	Digikey	WM8129-ND	90131-0131	CONN HEADER 22POS .100" STR TIN	2x11 expansion header	2.36	0	2.36	0
**General**	Top	P1	1	Digikey	609-3220-ND	67996-416HLF	CONN HEADER 16POS .100 STR TIN	2x8 male board 2 board header	0.83	0.277	0.83	0.277
**General**	Bot	P5	1	Digikey	WM4241-ND	901512116	C-GRID PCB CONN DR VT TN-A 16CKT	2x8 female board 2 board header	3.24	1.484	3.24	1.484
**General**			4				standoff	standoffs between boards	0.3	0.1	1.2	0.4
**General**			8				screws for enclosure		0.02	0.005	0.16	0.04
**General**			1				black foam for optical shielding in lid		0.04	0.04	0.04	0.04
										**TOTAL:**	**15.04**	**5.872**
**Manufacturing**			1	Adv. Ckits			PCB Fabrication		66	10	66	10
**Manufacturing**			1	Adv. Ckits			PCB Assembly (SMT soldering)		130.6	53.96	130.6	53.96
**Manufacturing**			1				3D printed enclosure (low quant)		10		10	0
**Manufacturing**			1	QuickParts			Injection molded enclosure (high quant)			2.5	0	2.5
**Manufacturing**			1				Assembly & Testing (low quant, 4 hr @ $20/hr)		80		80	0
**Manufacturing**			1				Assembly & Testing (high quant, 2hr @ $5/hr)			10	0	10
										**TOTAL:**	**286.6**	**76.46**

Manufacturing Totals		
	**Prototype**	**>5000**
**FULL BOX TOTAL**	**919.035**	**382.5966**
**NO LCD/GPS/CELL**	**553.916**	**223.7616**

Table S2

MicroBAR LAMP Reaction Cost Estimate

Reaction Volume: 6.28E-07 L
Reaction Cost: 0.012893607
Cost/uL: 0.020531221
Cost/chip 1.237786242

Solids

Reagent	Molecular Weight	Mass (g)	Moles	Cost	Cost/mole	Concentration/Rea	Moles/reaction	Cost/reaction	Fraction of Cost
Tris Buffer	127.2	100	0.786163522	$74	94.128	0.02	1.256E-08	1.18225E-06	0.01%
KCl	74.55	500	6.706908115	$31	4.59228	0.01	6.28E-09	2.88395E-08	0.00%
MgSO4.7H2O	246.47	500	2.02864446	$30	14.7882	0.008	5.024E-09	7.42959E-08	0.00%
(NH4)2SO4	132.14	100	0.756773119	$14	18.76388	0.01	6.28E-09	1.17837E-07	0.00%
MnCl2	197.91	100	0.505280178	$22	43.93602	0.001	6.28E-10	2.75918E-08	0.00%
Calcein	622.53	5	0.008031741	$103	12761.865	0.00005	3.14E-11	4.00723E-07	0.00%

Solutions

Reagent	Concentration (M)	Volume (L)	Moles	Cost	Cost/mole	Rxn Concentration	Moles/reaction	Cost/reaction	Fraction of Cost
Betaine	5	0.0015	0.0075	$20	2666.666667	0.8	5.024E-07	0.001339733	10.39%
dATP	0.1	0.00025	0.000025	$37	1470000	0.0014	8.792E-10	0.001292424	10.02%
dCTP	0.1	0.00025	0.000025	$37	1470000	0.0014	8.792E-10	0.001292424	10.02%
dGTP	0.1	0.00025	0.000025	$37	1470000	0.0014	8.792E-10	0.001292424	10.02%
dTTP	0.1	0.00025	0.000025	$37	1470000	0.0014	8.792E-10	0.001292424	10.02%
Bst Polymerase	8000000	0.001	8000	$244	0.0305	320000	0.20096	0.00612928	47.54%
Tween 20 (%)	1	100	100	$27	0.273	0.2	1.256E-07	3.42888E-08	0.00%
Primers FIP/BIP			0.0000005	45	90000000	0.0000032	2.0096E-12	0.000180864	1.40%
Primers LoopF/B			0.0000002	10	50000000	0.0000016	1.0048E-12	0.00005024	0.39%
Primers F3/B3			0.0000002	16	80000000	0.0000004	2.512E-13	0.000020096	0.16%

Reagents are from Sigma or New England Biosciences, Primers are from IDT

Figure S3

Disposable Microfluidic Cartridge Layout

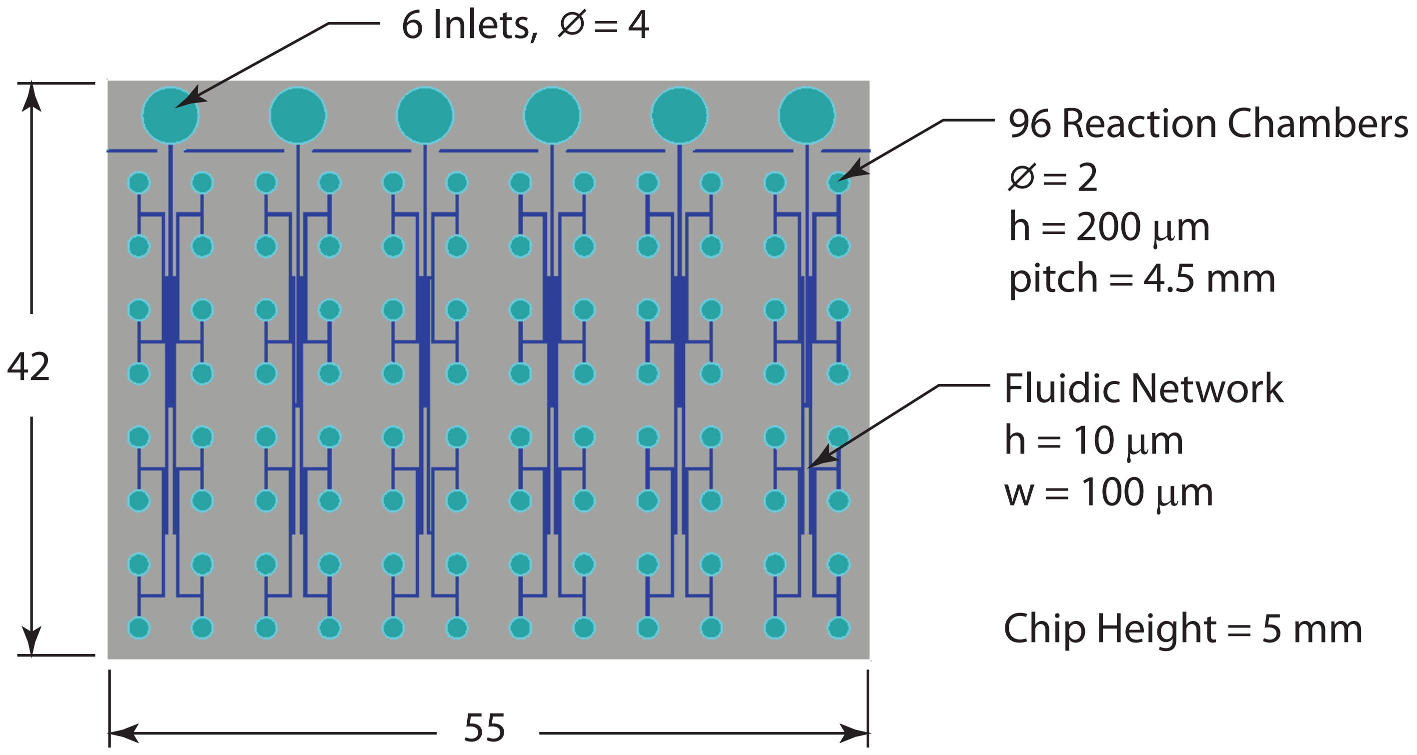

Dimensions in mm unless otherwise specified

Passive Degas-Driven Sample Loading

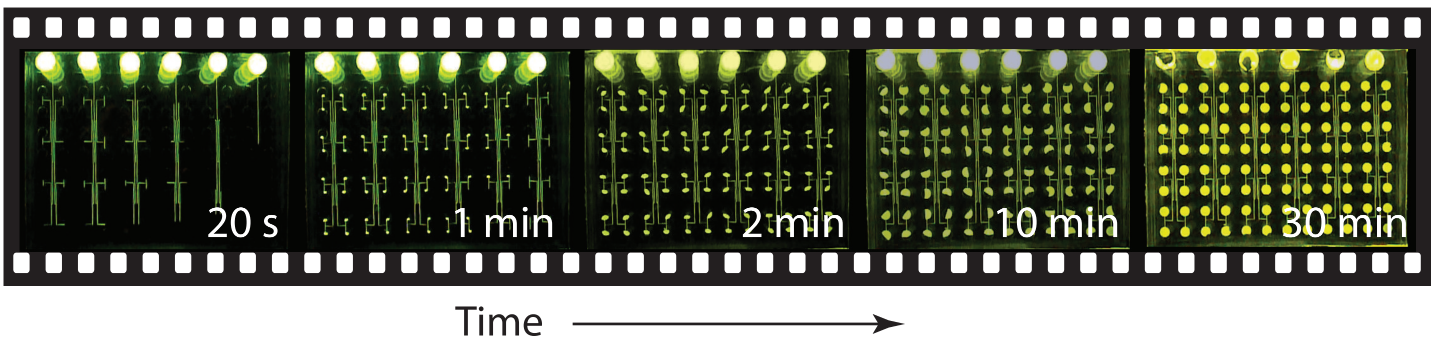

MicroBAR Assembly Procedure

Rev. 2013-06-21

1. Materials you need:
    a. PCBs (order from Advanced Circuits)
    b. SMT solder paste stencils (order from Pololu)
    c. All components listed in bill of materials
    d. Tube of solder paste
    e. Trowel (glass slide will also work)
    f. Skillet
    g. Fine tipped tweezers
    h. Tape and scrap PCBs
    i. Power supply with a cable to plug into JST battery input connector
    j. Conductive epoxy
    k. Spray paint and primer
    l. 2-part clear epoxy (fast drying)
    m. AVR-ISP mkII in-circuit programmer
2. Printing 3D enclosure
    a. Each piece is saved as a separate STL file. Load these all simultaneously into the Zprinter software and it should automatically arrange them. You can do a "fix" on the parts if there have been changes.
    b. 3D print using default settings
    c. After 24 hours or so, remove the parts and epoxy according to directions. Let sit overnight.
3. Tapping holes in 3D enclosure
    a. In current version, I drilled pilot holes (no actual tapping) with a #43 bit in the PCB pillars and in the lid. But the material strips easily with these fine threads. In the next version, there shouldn't be any tapping (except maybe the lid). Rather, just use pockets for nuts/4-40 sleeves in the lid and base. So in all places where there should be a nut (8 in the lid and 4 in the base) that is epoxied in.
    b. Countersink the heads so the lid closes flat (may also print that directly into future designs)
    c. It's good to test fit the components. The display required some tweaking
4. Painting 3D enclosure
    a. Sand the enclosure thoroughly with 100 grit sandpaper
    b. Prime, sand, prime, sand. Be careful of rounding off edges. I applied 2-3 coats of primer, but only a little at a time. So in total I probably sprayed it 8 times with primer, and 8 times with paint, considering I also had to rotate it and get it from all sides.
    c. Spray paint with semi-gloss black. Use thin coats and even strokes. No need to paint the interior, but definitely coat it with primer.
5. Populating PCBs (see Sparkfun tutorial on using Skillet for PCB reflow)
    a. Tape several scrap PCBs to the table around the PCB to be populated so it fits snug without sliding
    b. Align and tape the stencil over the PCB. The scrap PCBs are there so the trowel doesn't bow off the edges.
    c. Squeeze out *plenty* of solder paste at one end of the board.
    d. With one firm motion, smear the solder paste over the stencil, try to keep it very uniform and get paste in all the holes. Touchup if necessary
    e. Remove the stencil with one motion.
    f. Place all the components on the top side of the board. Ensure you've got the correct polarity on things. Most solder paste bridges will fix themselves, if there's not too much solder.
    g. Plug in the skillet and turn it all the way to max. Place the board on the skillet. The warmest region is around the circular heating element.
    h. After about 3 minutes, the paste will begin to reflow. Move the board around a bit if one part isn't getting hot enough. The whole process should take <5 minutes.

i. When all the components have reflowed, turn off the skillet and carefully remove the board and set it down to cool.
j. Inspect all components for solder bridges, bad connections, etc.
k. Flip the board over and populate it with a regular tip iron
l. Populate all through-hole components by hand. If populating the cell phone module, take special care that the 60-pin socket doesn't have any solder bridges and the module is soldered down to the board securely.
m. Populate the rectangular LEDs, with special care to bend them at the correct position and solder them at the correct height.
n. Snip off the tip of the pushbutton - this will make assembly easier (only one detent is needed)
o. Note the PCB mods that I made in the "readme.txt" file and make these revisions (mostly adding jumper wires). These should be incorporated into the next version.

6. Initial testing
    a. Plug a power supply with current limiting capability to the battery in port and turn it all the way down.
    b. Slowly ramp up the voltage on the power supply and make sure there isn't a significant current draw. If there is, you've likely got a solder bridge short.
    c. Set the power supply to 4 V and probe the 5V, 3.8V, and 3.3V supplies on the board to make sure they're correct. Vary the power input and make sure they are stable
    d. Turn off the power supply and connect the top board (screw in the standoffs). Repeat this procedure.
    e. Connect a LiPo battery to the port and connect the USB port to a computer. Verify that the USB port does not overdraw current and that the battery voltage increases steadily (charging) when it is plugged in. Also verify that when you press the power switch, the computer recognizes new hardware (Atmel 8U2)
    f. Dry fit the boards to the 3D enclosure (if available) and make any adjustments to the protruding components, if necessary.
7. Install battery and cell phone antenna
    a. Cut brackets out of brass for the cell phone antenna and the LiPo battery. Attach these to the bottom PCB and mount the antenna and battery. There should be a piece of foam between the battery and the bottom of the PCB to prevent the bottom components from digging into the battery.
8. Downloading the AVR firmware (USB controller and Arduino bootloader)
    a. Make sure drivers are installed for the AVR-ISP mkII (easiest way to do this is to install AVR Studio)
    b. Connect the AVR-ISP mkII to the ICSP2 header to download the 8U2 DFU firmware.
        i. Note that this firmware is the same as that available from the Arduino website, but I modified the descriptor string.
        ii. Download with:
        avrdude -p at90usb82 -F -P usb -c avrispmkii -U flash:w:MicroBAR_dfu_and_usbserial_combined.hex -U lfuse:w:0xFF:m -U hfuse:w:0xD9:m -U efuse:w:0xF4:m -U lock:w:0x0F:m
    c. Make sure the board enumerates as "Berkeley MicroBAR 2"
        i. Point Windows to the "Windows Driver" directory and install the driver
    d. Plug in the mkII to the ICSP1 header and fire up the Arduino software. Make sure the correct board (Arduino 2560) is selected and correct COM port is selected, then go to "burn bootloader"
    e. Unplug the mkII and power cycle the board.
    f. Load the Controller.ino software onto the MicroBAR
9. Firmware testing
    a. When the MicroBAR restarts, you should hear two beeps and see the RUN/BAT lights flash. This indicates that the firmware is running.
    b. Open the arduino serial monitor and verify that you get state data from the board. Then run the Processing UI and verify that you see data from the phototransistors and that you can tell the board to run an assay.

c. Verify that all 96 phototransistors respond individually (block out all but one using black foam and observe that each one is uniquely addressed). If there is a problem, it may indicate a solder bridge on the addressing pins.

10. Assembling ITO heater
    a. Start w/ 75x50x0.5mm ITO coated glass slide from Delta Technologies (5-15 ohm/sq)
    b. Verify which side is conductive
    c. Cut ITO heater brackets
        i. Start w/ ~100um brass bar from Ace HW, 3/4" wide, K&S Engineering 614121102330
        ii. Cut to width of slide w/ tin snips (50mm)
        iii. Mark 5mm line on one side
        iv. Use tin snips to cut the pins (about 1.5mm wide)
        v. Put in vice (pins down) and cut at 5mm line using Dremel cut-off wheel
        vi. Use wire brush wheel to remove flashing and roughen back side (where epoxy will go)
        vii. Put in vice and bend legs 90 degrees
        viii. Make sure pins will insert into PCB.
    d. Glue on brackets and thermistor
        i. Lay brackes face down on kimwipe
        ii. Dry fit slide, make sure spacing is correct, double check for conductive side
        iii. Before gluing the slide, put a dot where the thermistor will go (middle of slide ~5mm in)
        iv. Mix conductive epoxy and smear on bracket evenly. Cut a small piece of the brass to apply it to the slide.
        v. Lay slide down (conductive side facing down) and push IN on brackets a bit so excess epoxy goes outside rather than smearing on the slide. Make sure it makes contact along entire length of bracket. Let it sit with a light weight on the slide for 10 minutes or so.
        vi. Mix 2-part epoxy (Loctite quickset) and then pickup a small droplet (brass piece works for this) and apply on the slide.
        vii. Use tweezers to place thermistor on slide, in the middle of the droplet
        viii. Once glue has set (20 min) transfer to oven (60C) for 1 hr. Or let sit overnight.
11. Adding LEDs/waveguides
    a. Cut/spraypaint waveguides
        i. Cut (2) 50x75x1mm glass slides and make 2 50x7mm strips to use as waveguides. Best to cut each waveguide from a separate slide.
        ii. Use flat black spraypaint, lay waveguides on napkin flat, sandwiched between other glass slides to mask off the regions we don't want to spray paint. Best to use the slides you cut from, so the edge fits perfectly.
        iii. Shake can and spray evenly, lightly so it doesn't drip on the sides
        iv. Make sure to get both ends (small ends)
        v. Once one side's dry, flip them over and do the other
        vi. Use sandpaper if necessary to remove any paint on the clear sides
    b. Glue on waveguides
        i. Once spraypaint is dry (could put in oven to accelerate this), mix more 2-part epoxy and apply small droplets at the edges of the brackets.
        ii. Set the waveguide down on top of the brass brackets. Note that the glass should extend over the ITO a bit (to avoid scattering into the ITO) and should be flush at either end of the brass brackets.
        iii. Let glue dry
    c. Glue on LEDs
        i. Use helping hands to hold ITO slide vertically. Make sure the helping hands have lots of tape around them so they don't mark the ITO. Grip it by the brackets (not the waveguide).
        ii. Pull out 3 LEDs and line them up in the same orientation (you'll want to get them quickly)
        iii. Make sure all the polarities are the same (note the position of the green line on the back)
        iv. Mix more 2-pary epoxy and dip each LED into the epoxy (just barely, don't let the epoxy cover the contacts). Place them on the edge of the glass waveguide, 3 to a side. One should be in

the center and the other two should line up so they're between columns 3 and 4 on the chip (use a scrap chip to eyeball it)...or maybe measure this precisely.

  v. Once you've done 3, let the glue dry, flip over the ITO, and do the other side.
  vi. Make sure the polarities of the other side line up, so a series string around all 6 will have them all in the proper polarity.

d. Wire up LEDs
  i. Use soild wire wrap wire (32 awg) and cut small pieces to connect between the LEDs. Bend the ends of the wire at 90 degrees, and solder them on one by one. The end of one side will connect with the beginning of the other.
  ii. Use small stranded wire from ribbon cable (28awg) to make a connector tying the strings together and put a JST connector on it (make sure the orientation is correct).
e. Coat all LEDs/wires with liquid electrical tape and make sure no light can escape, except through the waveguide. Hold the slide such that whichever side you're currently coating is facing down, since the liquid electrical tape tends to drip. 3-4 coats seems to do the trick. Note that this is also strain relief for the leads.
f. Let it dry
g. Connect leads to thermistor. Use smallest heatshrink to protect leads from each other, and put a JST connector on the other end.

12. Plug in the ITO, thermistor, and LEDs and test them using the software. Check to make sure the ITO is getting hot, the LEDs can vary their brightness across all the levels, and the thermistor is reading approximately the correct temperature. The ITO should have approximately 10V across it. The LEDs should not flicker.
13. Cutting foam insert and gasket
  a. The thinner foam (1/16") is for the insert underneath the ITO, and the thicker foam (1/8") is for the light gasket.
  b. These burn very easily, so cut them on the lowest power setting on the Versalaser (Paper). I used ~ 0.1mm for the 1/16" @ +10% or so, and 1mm @0% for the gasket (both on Paper).
  c. Test fit both
14. Cut green gel to fit array and sandwich this between the ITO and foam. Get the height level and as close as possible to the PTs without the slide touching, then solder down the ITO.
15. Attaching light gasket
  a. Mix up more epoxy and smear it in the trench on the base, careful to avoid getting it around the sides. Insert the gasket. Do this quickly since the epoxy sets in 5 min. DO NOT put something heavy on the foam while it's drying or it will be permanently compressed.
16. Cutting battery and antenna brackets
  a. See current design. Antenna bracket is cut from brass, 9mm wide, with a 4-40 hole at one end and a 90 degree bend at the other for the SMA connector to thread through.
  b. Battery bracket should fit between the holes under the board and be a little larger than the battery, so foam will fit snugly in there.
  c. Attach the battery. Use a 4-40 nut/bolt. Cut a square of foam to go between the battery and PCB, and a strip of foam to go along the underside of the bracket.
  d. Thread the SMA connector into the bracket.
17. Installing PCBs
  a. In this version, the clearances really aren't sufficient. The enclosure should be enlarged slightly.
  b. Use a drill bit for 75% thread engagement for 4-40 screws and drill holes in the pillars. Note that this isn't ideal, and you should instead epoxy nuts in the pillars since the 3D printed material is prone to stripping.
  c. Screw in the top board using the mid-size standoffs from Digikey). Put the board in and use a 4-40 screw to actually drive the standoff into the material (let the screw bottom out in the standoff). Then hold the standoff with pliers and back the screw out.
  d. To get the bottom board in, make sure all the LEDs are bent so they will sit just behind the enclosure and bend the board-to-board header towards the hinge side so the pins are at an angle. Then you can wiggle the bottom board in and get it to mate while the USB port protrudes through. Pushing

the switch in the ON position helps.

   e. Screw in 4 more standoffs (same length). Make sure to attach the antenna bracket to one of them. This is what the base will screw in to.

18. Installing LCD module
   a. Burn LCD firmware on via USB port
   b. Modifications to Amulet LCD
      i. Remove RS-232 connector and power connector. I just used angle cutters to cut them apart and then desoldered the pieces from the board
      ii. Cut the traces which go to the MAX232 on the board (these are clearly marked)
      iii. Solder the TX/RX/+5/GND leads onto the board directly. The wire should be ~8" long
   c. New enclosure version should have pillars for mounting the LCD with embedded 4-40 nuts. So you'll just screw it in. I just cut foam (2 thick pieces) to sandwich the display in. I cut out holes in the foam for all the components on the PCB.
19. Installing GPS module
   a. Use double sided tape or hot glue to glue the module into the lid.
   b. Make a small JST connector w/ 5 wires coming off it.
   c. Attaching lid pieces and verifying that hinge works
   d. Use hot glue to attach the GPS receiver. The LCD can be floating (next version it should fit more snugly).
   e. Thread the GPS/LCD cables through the hinge and put the hinge together
   f. Screw the hinge together using wood screws (#4, 5x8")
   g. Add JST connectors to GPS cable and LCD cable.
   h. Plug GPS/LCD into PCB.
20. Attaching base
   a. Screw it in using 3/4" 4-40 screws. These should be a little more than finger tight...any more and you will strip out the pillars.

Text S2

MicroBAR Operating Instructions

Rev. 2013-06-21

Making PDMS Chips

1) The chip mold is fabricated using standard SU-8 lithography techniques
2) Chips are molded from Sylgard 184 PDMS with a 10:1 curing weight ratio
3) After molding, remove the chips from the wafer and cut along the edges with a sharp straight razor blade. Note that initially we cut a little beyond the edge of the chip and we make the final edge cut after the "floor" of the chip is bonded.
4) A 4 mm hole punch is used to create the inlet wells (Harris Unicore, Ted Pella). Make sure to clean the punch with Eliminase.
5) The bottom of the chip is cut from Stockwell Elastomerics .010" Thick HT6240 sheets.
6) Both the PDMS channels and the "floor" sheet are treated with Eliminase to remove DNA, DNAse, and RNAse and then are rinsed with ultrapure water.
7) The two chip parts are bonded using oxygen plasma.
8) After bonding, the edges are cut to the precise dimensions

Setting up a Chip

1) Currently, the LAMP reaction mix is prepared off-chip under sterile conditions. Template is added last.
2) Positive and negative reaction mixes are split into two tubes: one for the thermocycler and one for the MicroBAR
3) The MicroBAR cartridge is vacuum treated for > 1 hr prior to loading.
4) At this point, cartridges may be sealed for future use using a commercial food sealer (although they should be vacuum treated first with a more powerful vacuum as the food sealer isn't powerful enough to enable full vacuum loading).
5) 20 uL of each reaction mix is loaded into each inlet immediately after it is taken out of vacuum. It takes about 30 minutes for the reaction mix to fully load into all chambers.

Operating the MicroBAR

1) Make sure you have a fully-charge battery or you have the instrument plugged in to wall power (note that we added a power plug in lieu of the LiPo battery so that the instrument could be powered continuously with a 5V wall-wart power supply. The power supply you use should have a rating of at least 2A.
2) During powerup, you should see the lights go through all 16 brightness levels, the run and bat lights flash once, and you should hear two beeps.
3) Plug the box into the USB port
4) Fire up the UI
5) Often, it doesn't connect the first time. Close the UI, unplug the USB and plug it back in again. That almost always fixes it.
6) Set your assay parameters (note that we almost always use the defaults, which are given in parentheses):
   a. Step time (2000 ms)
      The time between samples. Note that internally, the MicroBAR averages 100 samples per sample that it returns.
   b. Stabilization steps (10)
      The photodetector amplifier needs a little time to reach a steady state value, so we let the instrument run for a certain number of steps before we start recording.
   c. Run steps (5000)
      This determines the length of the assay. Run steps * step time = total assay time (although actually it's a little off due to the way it calculates things).
   d. Temp (60 C)
      This is the run temperature
   e. LEDs (1)

Sets the light level. These are logarithmically spaced intensity values from 1-15 (0 is OFF, 1 is lowest intensity). Values above 1 tend to saturate the detectors, so we stick with 1.

f. Start Well (1)

If you only want to sample a subset of wells, you specify the start and stop here.

g. End Well (96)

Stop well to sample.

7) Click "Set Assay Params" and verify that the unit responds with the correct parameters (the unit's state is printed on the right side)
8) Set the appropriate output directory. Note that it must already exist. Don't include a filename here, that will be automatically added (.log)
9) Insert chip and line it up with the phototransistors. Make sure that the bottom of the chip sits flat and the PDMS "wets" the surface of the ITO/glass slide. This will ensure that it doesn't flex during heating.
10) Close the lid gently and make sure it completely seals (might have to wiggle it back and forth a bit)
11) Click Start Assay and wait
12) The unit will emit 3 long beeps when it's done
13) To view the data, load the Matlab script and change the filename to your log file. It takes a long time to parse the log files, but it saves them as a MAT file so you can do this again and it's much faster.
14) Specify the colors of the wells you want to plot. PLOT_COLORS tells the script what color to print (standard matlab 1-letter abbreviations). If you set a well to 0, it won't print.

Summary of Files Available on GitHub

Rev. 2013-06-21

The GitHub repository contains all of the design and source code files for the MicroBAR in their native format. These include:

Schematics and PCB Layout
Software: Altium Designer 10
This folder includes two separate projects for the two boards in the MicroBAR:
1. LAMP Chip PCB.PrjPCB
2. LAMP Microcontroller PCB.PrjPCB

Microcontroller and LCD Firmware
Software: Adruino 1.0.4 and Amulet Technologies GEMcompiler 1.1.2.
This folder includes:
1. Amulet LCD GUI. The "mainmenu.htm" is the root page that should be downloaded to the LCD.
2. Atmel8U2 DFU USB Firmware. This should be programmed onto the 8U2 USB interface chip via AVR-ISP.
3. Arduino Sketches. These include the firmware code for the MicroBAR. The "controller.ino" is the root sketch. Code for hardware peripherals (LCD, Header, Lights, GPS, etc.) is included in separate sketch files.

Enclosure CAD Files
Software: Rhinoceros 4
This folder includes the original enclosure design (enclosure.3dm) as well as exported STL files for 3D printing.

PC Control and Analysis Software
Software: Processing 1.2.1 and MATLAB
This folder includes:
1. Windows Driver. Once the 8U2 firmware is loaded, Windows will be able to use this driver to communicate with the MicroBAR as a serial port.
2. Processing UI. This is the main control program for the MicroBAR, written in the Processing language. LAMP_UI.pde is the main file.
3. MATLAB Analysis Scripts. All analysis and data visualizationwas carried out in MATLAB. plot_data_from_log.m parses log files generated by the Processing UI and generates a series of plots. microbar_calibration.mat includes the sensitivity coefficient matrix for the detector array.

Microfluidic Chip Layout
Software: AutoCAD 2012
This folder includes the standard chip layout that we use in this manuscript, which features 96 reaction wells at 4.5 mm pitch, with 16 wells connected in parallel to each of 6 inlets.
